# Antifungal-induced DNA dynamics and chitin remodelling across *Cryptococcus* spp. and the novel broad-spectrum anti-cryptococcal candidate CPTH2

**DOI:** 10.1038/s41598-026-52566-9

**Published:** 2026-05-19

**Authors:** Diana Tamayo, Rahul Anand, Nicolas Helmstetter, David M. Engelthaler, Matthew C. Fisher, Robin C. May, Rhys A. Farrer

**Affiliations:** 1 Medical Research Council Centre for Medical Mycology, Department of Biosciences, Faculty of Health and Life Sciences, University of Exeter, Exeter, EX4 4QD UK; 2 Arizona State University, Tempe, USA; 3 Translational Genomics Research Institute, Flagstaff, AZ USA; 4 MRC Centre for Global Infectious Disease Analysis, Imperial College School of Public Health, Imperial College London, London, W12 0BZ UK; 5 Institute of Microbiology and Infection, School of Biosciences, University of Birmingham, Birmingham, B15 2TT UK

**Keywords:** *Cryptococcus*, Antifungals, Resistance, 5-fluorocytosine, Fluconazole, Amphotericin B, Epigenetic inhibitors, CPTH2, Phenotypic screening, DNA dynamics, Chitin remodelling, Multivariate analysis, Drug discovery, Microbiology

## Abstract

Antifungal drug resistance is a growing global health concern, yet treatment options remain limited to just four classes: polyenes, azoles, allylamines, and echinocandins. *Cryptococcus* species are responsible for life-threatening meningoencephalitis and pulmonary infections, which cause over 140,000 deaths annually. The high mortality of *Cryptococcus* is driven by limited drug access, toxicity, persistence, and emerging resistance. To better understand antifungal resistance and identify novel therapeutics, we profiled a genetically diverse panel of *Cryptococcus* isolates spanning all major lineages (VNI, VNIII, VNIV, VGI–VGVI). Phenotypic assays were used to assess susceptibility to fluconazole (FLZ), 5-fluorocytosine (5-FC), and amphotericin B (AmpB), as well as the epigenetic inhibitors CPTH2 (a histone acetyltransferase inhibitor) and SAHA (a histone deacetylase inhibitor). We found lineage- and strain-specific variation in antifungal susceptibility, with disc-diffusion and microdilution assays yielding consistent patterns. Pharmacodynamic modelling revealed potent fungicidal activity of CPTH2 across all tested lineages. DNA content and chitin levels varied significantly by lineage and treatment: FLZ and CPTH2 induced DNA content increases in susceptible strains, suggesting a stress response, while 5-FC-induced chitin remodelling was most pronounced in resistant strains. Multivariate analysis identified chitin induction as a principal driver of phenotypic variance under antifungal stress. Our findings reveal lineage-specific antifungal responses in *Cryptococcus*, highlight the antifungal potential of epigenetic inhibitors such as CPTH2, and underscore the utility of phenotypic profiling to inform drug development and resistance surveillance.

**Supplementary Information:**

The online version contains supplementary material available at 10.1038/s41598-026-52566-9.

## Introduction

Antifungal drug resistance is an escalating public health concern, yet treatment options remain limited to just four drugs classes^1^. Unlike antibiotics, antifungals are hindered by high host toxicity, emerging resistance, and narrow-spectrum activity, leaving clinicians with few safe and effective choices. While advances such as liposomal amphotericin B (AmpB) formulations have reduced toxicity, the identification of novel antifungal targets that are both potent and host-safe remains a critical unmet need. Resistance evolves rapidly through mechanisms including point mutations, altered gene expression, aneuploidy, and epigenetic regulation^2,3^. This problem is exemplified by *Cryptococcus* species which are intrinsically resistant to echinocandins, which is one of the safest antifungal classes, underscoring the need for new therapeutic strategies^4^.

*Cryptococcus neoformans* and *Cryptococcus gattii* cause cryptococcosis, a life-threatening fungal infection that predominantly affects the lungs and central nervous system (CNS). Dissemination to the CNS results in cryptococcal meningitis (CM), a major cause of morbidity and mortality in immunocompromised individuals. These genetically diverse species complexes comprise ten distinct phylogenetic lineages: *C. neoformans* (var. neoformans) includes four lineages (VNI-VNIV), while *C. gattii* includes six (VGI-VGVI)^5,6^. *C. neoformans* lineages cause most systemic cryptococcosis cases, particularly CM in individuals with HIV/AIDS, whereas *C. gattii* lineages more commonly infect immunocompetent hosts, reflecting distinct ecological and evolutionary trajectories^7–10^.

CM remains the most common cause of fungal meningitis worldwide, responsible for an estimated 194,000 cases and 147,000 deaths annually^11^. Regardless of genotype, standard therapy involves induction with liposomal AmpB and flucytosine (5-FC), followed by fluconazole (FLZ) consolidation. However, both AmpB and 5-FC are associated with severe toxicities, including nephrotoxicity (AmpB) and hepatotoxicity or bone-marrow suppression (5-FC)^12,13^. Drug access remains poor in heavily affected regions, including sub-Saharan Africa and parts of South America, including Bolivia, Venezuela, Ecuador, Chile, and Suriname^14–17^. Even with optimal therapy, mortality rates often exceeding 75% in low- and middle-income countries (LMIC)^11,18–20^, reflecting barriers including limited drug access, high host toxicity, incomplete understanding of resistance mechanisms, and emerging resistance. Although a single high-dose liposomal AmpB regimen has shown promise in lowering mortality to approximately 30%, its high cost and limited distribution continue to restrict widespread implementation^21,22^.

Fungi possess plastic genomes that tolerate genomic copy number variation (CNV) caused by aneuploidy, gene amplification, or deletion, enabling adaptation to stressful conditions, including antifungal exposure^23,24^. Azole treatment induces aneuploidy and polyploidy in fungal pathogens, including *Cryptococcus* and *Candida* species, with FLZ-resistant *C. neoformans* strains exhibiting disomies of chromosomes 1, 4, 10, and 14^25,26^. Antifungal-induced genomic changes are not just a passive consequence of stress, but an active adaptive mechanism^23^. Chitin remodelling also mediates antifungal stress responses, particularly during echinocandin exposure. The fungal cell wall is a dynamic structure, and increased chitin deposition can compensate for cell wall damage caused by antifungals^27^. Flow cytometry enables detection of drug-induced DNA- and chitin- content changes as a proxy for ploidy shifts or chitin remodelling, respectively. While DNA content changes following FLZ exposure have been documented in *C. neoformans*^25^, comparable data for 5-FC or AmpB do not exist, nor have such responses been systematically evaluated across *Cryptococcus* lineages.

Epigenetic regulators represent promising antifungal targets. Chromatin-modifying enzymes including histone methyltransferases (HMTs), histone acetyltransferases (HATs) and histone deacetylases (HDACs) play essential roles in fungal stress responses, immune evasion and antifungal drug resistance in diverse pathogenic species^28–30^. These enzymes influence transcriptional plasticity under drug pressure and environmental stress, making them attractive intervention points to overcome limitations of current antifungal agents, including toxicity, resistance, and narrow spectrum of activity. Among these, HAT inhibition (HATi) has shown particular promise. For example, the HATi CPTH2 has demonstrated antifungal activity against the *Candida* CTG clade and multidrug resistant *C. auris*, where either depletion of the HAT *GCN5* or its pharmacological inhibition with CPTH2 impaired ergosterol biosynthesis and efflux pump expression, increasing susceptibility to azoles and polyenes. Notably, CPTH2 synergised with caspofungin in vitro and in vivo, without notable host toxicity^30,31^. These findings establish CPTH2 as a compelling candidate for further evaluation in *Cryptococcus.*

Here, we performed phenotypic screening across a genetically diverse panel of *Cryptococcus* strains representing all major *C. neoformans* and *C. gattii* lineages (VNI, VNIII, VNIV, VGI–VGVI). Our aim was to determine whether antifungal treatment alters DNA and chitin content across this genetic diversity, and whether these responses differ between strains with varying susceptibility profiles. We tested standard agents (5-FC, FLZ and AmpB), the HATi CPTH2, and the HDAC inhibitor (HDACi) suberoylanilide hydroxamic acid (SAHA). For initial screening, single concentrations were selected based on tentative EUCAST-based epidemiological cutoff values (ECVs) reported previously^32,33^, while higher exploratory concentrations were used for epigenetic inhibitors to ensure detection of activity. SAHA displayed no antifungal activity and no significant DNA and chitin content changes, while CPTH2 demonstrated broad antifungal properties coinciding with changes in DNA content. We further assessed the antifungal potential of CPTH2 through a drug-response modelling in three representative lineages (VNI, VGI and VGII). Phenotypic parameters included relative growth, chitin and DNA content, and inhibition zone diameter. Our panel included 2–3 representative strains per lineage; larger strain sets will be necessary to determine whether the patterns observed here are broadly generalisable across each lineage or reflects strain-specific variation. Overall, our findings highlight the potential of CPTH2 as a broad-acting-anti-cryptococcal agent, while also providing new insight into strain-specific variation in DNA and chitin responses to antifungal stress. These phenotypic markers may inform future resistance surveillance strategies and provide a foundation for mechanistic studies linking genomic plasticity and cell wall remodelling to antifungal tolerance in *Cryptococcus*.

## Results

### Epigenetic inhibition with CPTH2 overcomes strain-specific variation in antifungal susceptibility in *Cryptococcus*

**Fig. 1:**
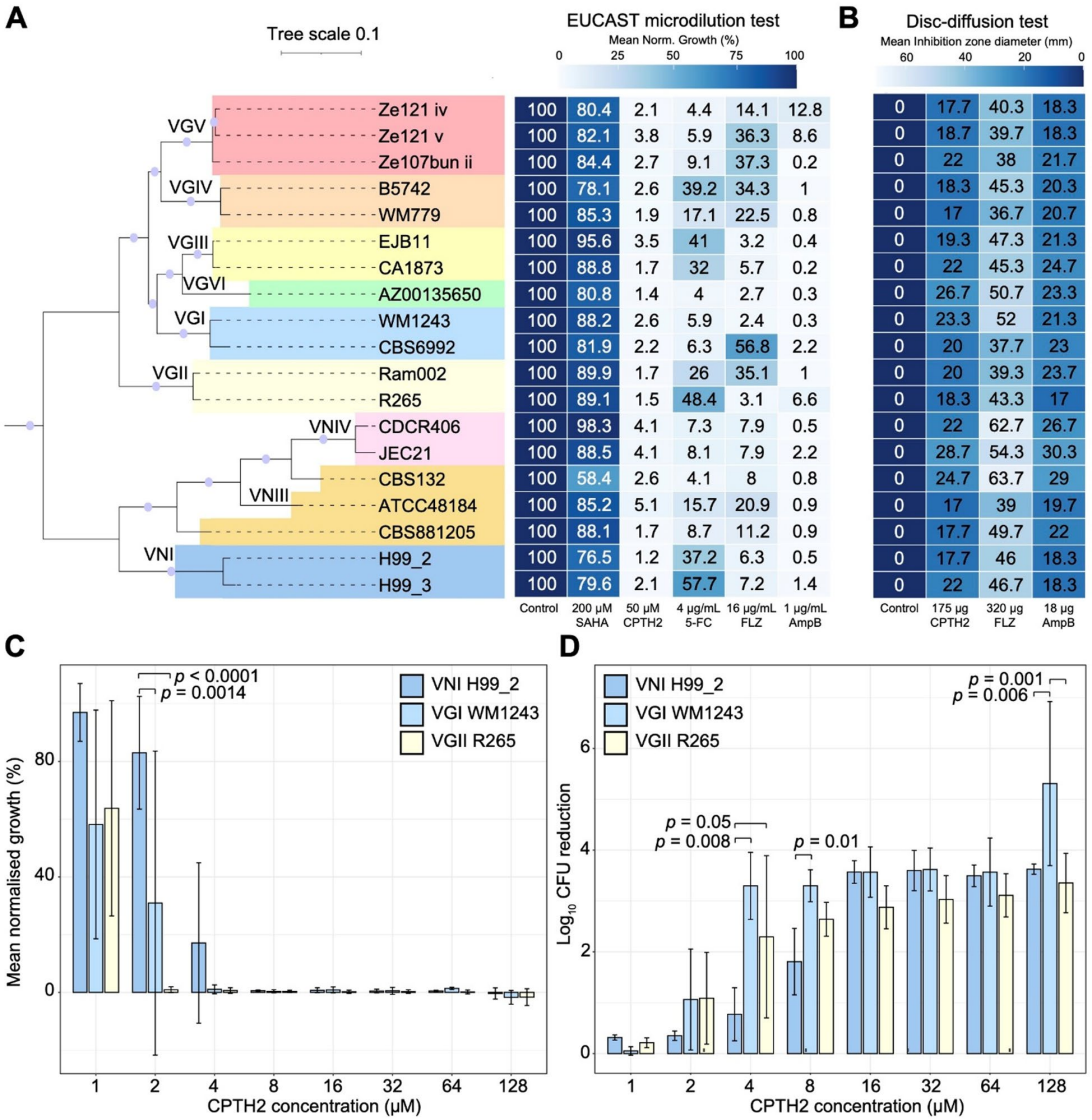
Susceptibility of *Cryptococcus* lineages to different antifungal agents. **(A) (Left)** Phylogenetic tree constructed using RAxML (GTRCAT model, 1000 bootstrap replicated) from 6967 nuclear sites covered of all 19 isolates, comprising of 12 *C. gattii* and 7 *C. neoformans* genomes. Branch lengths indicate the mean number of changes per site. **(Right)** Adjacent heatmap displays the mean relative growth of each strain following exposure to the compounds, expressed as percentage of growth relative to untreated controls. Color intensity reflects growth levels (dark blue = higher growth, lighter blue = reduced growth), with a scale bar indicating the percentage range. **(B)** Heatmap of inhibition zone diameters measured by disc-diffusion assay. Color intensity corresponds to the size of the inhibition zone (dark blue = smaller zone (less susceptible), light blue = larger zone (more susceptible)). Numerical values inside each cell represent the mean of three independent biological replicates (± SD). Strains are ordered based on their phylogenetic relationships. **(C)** Bar plots showing the percent growth and **(D)** log_10_ reduction in CFUs for three representative strains: VNI H99_2, VGI WM1243, and VGII R265, exposed to a two-fold dilution series of CPTH2. Experiments were performed in three independent biological replicates (± SD). Statistical analysis was performed using a two-way ANOVA model (Inhibition ~ Strain x Concentration), followed by pairwise comparisons using emmeans at each concentration.

Standard antifungals showed variable and generally limited activity that depended on *Cryptococcus* genotype, whereas the HATi CPTH2 demonstrated broad and consistent activity across lineages. Across strains, 5-FC and FLZ were the least active (relative growth ranges from 3.96 to 57.7% and from 2.41 to 56.8%, respectively), AmpB was the most active among the standard drugs (0.17–12.85% growth), and CPTH2 produced consistently strong growth inhibition (1.23–5.15% growth). SAHA had minimal antifungal activity, with relative growth near 98% (Fig. 1A).

For 5-FC, 9/19 strains (~ 47%) showed decreased susceptibility, spanning *C. gattii* VGII, VGIII, VGIV and in *C. neoformans* VNI (**Figure S1a**). Increased susceptibility was observed only in two strains, VGVI AZ00135650 and VNIII CBS132. For FLZ, 7/19 strains (~ 42%) were classified as decreased susceptibility, mostly within *C. gattii* complex (6/12) (**Figure S1b**). This was particularly evident in VGV (Ze121V, Ze107bunii), VGIV (B5742, WM779), VGI (CBS6992) and VGII (Ram002). In contrast, VGVI AZ00135650 and VGI WM1243 were classified as increased susceptibility, while several *C. neoformans* strains were indeterminate. Only one *C. neoformans* strain, VNIII ATCC48184, was classified as decreased. These patterns suggest inter-lineage differences (e.g., VGV/VGIV vs. VGVI) and intra-lineage variation (e.g., VGI: WM1243 increased vs. CBS6992 decreased; VGII: Ram002 decreased vs. R265 near the increased boundary (**Figure S1b**)).

For AmpB, decreased susceptibility was limited to three strains (16%): VGII R265 and VGV Ze121V/Ze121IV (**Figure S1c**). All others were strongly inhibited (< 3% relative growth). By contrast, CPTH2, showed uniformly high activity, with no strains meeting the criteria for increased or decreased susceptibility (growth 1.23–5.15%, ~ 95–99% inhibition) (**Figure S1d**). SAHA, included as an epigenetic control, showed no antifungal effect but allowed us to separate antifungal activity from potential DNA or chitin related responses to treatment.

Taken together, these results suggest (*i*) inter-lineage differences in response to FLZ and 5-FC, (*ii*) intra-lineage heterogeneity (notably in VGI and VGII), and (*iii*) the consistently strong activity of CPTH2 across the *Cryptococcus* genus at 50 µM.

### Disc-diffusion and microdilution tests for FLZ show strong correlation in detecting strain susceptibility differences

To further evaluate antifungal susceptibility, we used in-house disc-diffusion assays for FLZ (320 µg), AmpB (18 µg) and CPTH2 (175 µg) (Fig. 1B). FLZ inhibition zones ranged from 36.7 to 63.7 mm, with ~ 26% of strains showing decreased susceptibility, largely concordant with microdilution data (Table 1). VGV Ze121V and VGIV B5742 showed decreased susceptibility only by microdilution, whereas VNIV (CDCR406 and JEC21) and VNIII CBS132 were classified as increased only by disc-diffusion. A significant correlation between both assays (Pearson’s *r* = 0.60, *p =* 0.0062; **Figure S2a**), supports the robustness of both methods in assessing FLZ susceptibility in *Cryptococcus*.

**Table 1:** Classification of *Cryptococcus* strains based on robust *z*-scores. Strain susceptibility classification based on robust *z*-scores from broth microdilution (*z* ≥ 0.8; ≥ 5%) and disc diffusion (*z* ≥ 0.7; ≥ 3 mm) assays. Strains are ordered according to their phylogenetic relationships.

Strain	Lineage	Mean inhibition (%)	z-score	Classification	Mean inhibition area (mm)	z-score	Classification
		**Broth microdilution assay 5-FC**					
Ze121IV	VGV	95.64	0.62	Indeterminate			
Ze121V	VGV	94.10	0.42	Indeterminate			
Ze107bunii	VGV	90.85	0.00	Indeterminate			
B5742	VGIV	60.83	-3.90	Decreased susceptibility			
WM779	VGIV	82.93	-1.03	Decreased susceptibility			
EJB11	VGIII	59.00	-4.14	Decreased susceptibility			
CA1873	VGIII	67.98	-2.97	Decreased susceptibility			
AZ00135650	VGVI	96.04	0.67	Increased susceptibility			
WM1243	VGI	94.07	0.42	Indeterminate			
CBS6992	VGI	93.74	0.38	Indeterminate			
Ram002	VGII	74.02	-2.19	Decreased susceptibility			
R265	VGII	51.55	-5.10	Decreased susceptibility			
CDCR406	VNIV	92.69	0.24	Indeterminate			
JEC21	VNIV	91.90	0.14	Indeterminate			
CBS132	VNIII	95.94	0.66	Increased susceptibility			
ATCC48184	VNIII	84.30	-0.85	Decreased susceptibility			
CBS881205	VNIII	91.33	0.06	Indeterminate			
H99_2	VNI	62.81	-3.64	Decreased susceptibility			
H99_3	VNI	42.27	-6.31	Decreased susceptibility			
		**Broth microdilution assay CPTH2**			**Disc diffusion assay CPTH2**		
Ze121IV	VGV	97.95	0.21	Indeterminate	17.67	-0.79	Indeterminate
Ze121V	VGV	96.16	-2.15	Indeterminate	18.67	-0.45	Indeterminate
Ze107bunii	VGV	97.29	-0.66	Indeterminate	22.00	0.67	Indeterminate
B5742	VGIV	97.39	-0.52	Indeterminate	18.33	-0.56	Indeterminate
WM779	VGIV	98.15	0.48	Indeterminate	17.00	-1.01	Decreased susceptibility
EJB11	VGIII	96.52	-1.68	Indeterminate	19.33	-0.22	Indeterminate
CA1873	VGIII	98.31	0.69	Indeterminate	22.00	0.67	Indeterminate
AZ00135650	VGVI	98.55	1.01	Indeterminate	26.67	2.25	Increased susceptibility
WM1243	VGI	97.41	-0.50	Indeterminate	23.33	1.12	Increased susceptibility
CBS6992	VGI	97.79	0.00	Indeterminate	20.00	0.00	Indeterminate
Ram002	VGII	98.30	0.67	Indeterminate	20.00	0.00	Indeterminate
R265	VGII	98.49	0.94	Indeterminate	18.33	-0.56	Indeterminate
CDCR406	VNIV	95.94	-2.44	Indeterminate	22.00	0.67	Indeterminate
JEC21	VNIV	95.85	-2.55	Indeterminate	28.67	2.92	Increased susceptibility
CBS132	VNIII	97.44	-0.46	Indeterminate	24.67	1.57	Increased susceptibility
ATCC48184	VNIII	94.85	-3.88	Indeterminate	17.00	-1.01	Decreased susceptibility
CBS881205	VNIII	98.26	0.62	Indeterminate	17.67	-0.79	Indeterminate
H99_2	VNI	98.77	1.31	Indeterminate	17.67	-0.79	Indeterminate
H99_3	VNI	97.86	0.10	Indeterminate	22.00	0.67	Indeterminate
		**Broth microdilution assay FLZ**			**Disc diffusion assay FLZ**		
Ze121IV	VGV	85.86	-0.79	Indeterminate	40.33	-0.60	Indeterminate
Ze121V	VGV	63.72	-3.62	Decreased susceptibility	39.67	-0.67	Indeterminate
Ze107bunii	VGV	62.74	-3.74	Decreased susceptibility	38.00	-0.87	Decreased susceptibility
B5742	VGIV	65.72	-3.36	Decreased susceptibility	45.33	0.00	Indeterminate
WM779	VGIV	77.46	-1.86	Decreased susceptibility	36.67	-1.03	Decreased susceptibility
EJB11	VGIII	96.76	0.61	Indeterminate	47.33	0.24	Indeterminate
CA1873	VGIII	94.30	0.29	Indeterminate	45.33	0.00	Indeterminate
AZ00135650	VGVI	97.27	0.67	Increased susceptibility	50.67	0.63	Increased susceptibility
WM1243	VGI	97.59	0.72	Increased susceptibility	52.00	0.79	Increased susceptibility
CBS6992	VGI	43.21	-6.24	Decreased susceptibility	37.67	-0.91	Decreased susceptibility
Ram002	VGII	64.93	-3.46	Decreased susceptibility	39.33	-0.71	Decreased susceptibility
R265	VGII	96.90	0.63	Indeterminate	43.33	-0.24	Indeterminate
CDCR406	VNIV	92.10	0.01	Indeterminate	62.67	2.06	Increased susceptibility
JEC21	VNIV	92.10	0.01	Indeterminate	54.33	1.07	Increased susceptibility
CBS132	VNIII	91.99	0.00	Indeterminate	63.67	2.18	Increased susceptibility
ATCC48184	VNIII	79.11	-1.65	Decreased susceptibility	39.00	-0.75	Decreased susceptibility
CBS881205	VNIII	88.84	-0.40	Indeterminate	49.67	0.52	Indeterminate
H99_2	VNI	93.74	0.22	Indeterminate	46.00	0.08	Indeterminate
H99_3	VNI	92.78	0.10	Indeterminate	46.67	0.16	Indeterminate
		**Broth microdilution assay AmpB**			**Disc diffusion assay AmpB**		
Ze121IV	VGV	87.15	-15.08	Decreased susceptibility	18.33	-0.87	Decreased susceptibility
Ze121V	VGV	91.42	-9.71	Decreased susceptibility	18.33	-0.87	Decreased susceptibility
Ze107bunii	VGV	99.79	0.82	Indeterminate	21.67	0.10	Indeterminate
B5742	VGIV	99.04	-0.13	Indeterminate	20.33	-0.29	Indeterminate
WM779	VGIV	99.15	0.01	Indeterminate	20.67	-0.19	Indeterminate
EJB11	VGIII	99.65	0.64	Indeterminate	21.33	0.00	Indeterminate
CA1873	VGIII	99.83	0.87	Indeterminate	24.67	0.96	Increased susceptibility
AZ00135650	VGVI	99.70	0.70	Indeterminate	23.33	0.58	Indeterminate
WM1243	VGI	99.68	0.68	Indeterminate	21.33	0.00	Indeterminate
CBS6992	VGI	97.79	-1.70	Indeterminate	23.00	0.48	Indeterminate
Ram002	VGII	99.00	-0.18	Indeterminate	23.67	0.67	Indeterminate
R265	VGII	93.41	-7.21	Decreased susceptibility	17.00	-1.25	Decreased susceptibility
CDCR406	VNIV	99.54	0.50	Indeterminate	26.67	1.54	Increased susceptibility
JEC21	VNIV	97.79	-1.70	Indeterminate	30.33	2.60	Increased susceptibility
CBS132	VNIII	99.17	0.04	Indeterminate	29.00	2.22	Increased susceptibility
ATCC48184	VNIII	99.11	-0.04	Indeterminate	19.67	-0.48	Indeterminate
CBS881205	VNIII	99.14	0.00	Indeterminate	22.00	0.19	Indeterminate
H99_2	VNI	99.50	0.45	Indeterminate	18.33	-0.87	Decreased susceptibility
H99_3	VNI	98.60	-0.67	Indeterminate	18.33	-0.87	Decreased susceptibility

For AmpB, inhibition zones ranged from 17 to 30.3 mm, with ~ 26% of strains showing decreased susceptibility. Both assays identified VGII R265 and VGV Ze121IV/Ze121V as decreased, but VNI strains were classified as decreased only by disc-diffusion. This method also detected increased susceptibility in VGIII CA1873, VNIV CDCR406/JEC21, and VNIII CBS132.

In contrast, for CPTH2, the relationship between the two assays was weak (Pearson’s *r* = 0.10, *p* = 0.66; **Figure S2c**), primarily reflecting methodological differences rather than inconsistency. The disc-diffusion assay revealed a broader range of inhibition values (17–28.7 mm) and was more sensitive in distinguishing strain-specific responses, identifying both decreased (VGIV WM779 and VNIII ATCC48184) and increased susceptibility (VGVI AZ00135650, VGI WM1243, VNIII CBS132 and VNIV JEC21) (Table 1). These differences likely arise from the intrinsic characteristics of each method, such as drug diffusion and agar interactions. Together, the two approaches provide complementary perspectives on CPTH2 activity, improving detection of phenotypic variability.

### Pharmacodynamic characterisation reveals fungicidal potential of CPTH2 against major lineages driving the cryptococcosis burden

To address the limitations of conventional susceptibility testing and gain deeper mechanistic insights into the antifungal activity of CPTH2, we employed pharmacodynamic modelling to quantify its efficacy with greater precision. We selected three representative *Cryptococcus* strains: VNI H99_2, VGI WM1243, and VGII R265. These lineages account for the majority of global cryptococcosis cases^17,34^. This approach enabled a more rigorous assessment of CTPH2’s antifungal potential compared to standard disc-diffusion or microdilution methods.

CPTH2 demonstrated a dose-dependent inhibitory effect on *Cryptococcus* growth across the three linages, with near complete inhibition (≥ 99%) at higher doses (Fig. 1C). Dose-response modelling (LL.3 and LL.4) showed consistently low ED_50_ values (1.07 to 2.88 µM), highlighting CPTH2’s potent anti-cryptococcal activity (Table 2, **Figure S3a**). At 2 µM, VNI H99_2 was significantly less inhibited than VGI WM1243 and VGII R265 (52.1% and 82.1% less, respectively; *p* = 0.0014 and < 0.0001), although these differences decreased at higher concentrations, where CPTH2 suppressed all strains equally. These results suggest greater sensitivity of VGII R265 and VGI WM1243 to CPTH2 at low concentrations.

**Table 2:** Pharmacodynamic modelling of CPTH2 across three lineages. Modelling parameters for CPTH2-induced growth inhibition: LL.4 model was applied for VNI H99_2 and VGI WM1243. LL.3 modelling was fitted for VGII R265. ED_50_ and ED_90_ values are shown with 95% confidence intervals (CI).

	VNI H99_2	VGI WM1243	VGII R265^d^
**Slope** ^a^	-4.76 (*p* = 0.0009)	-5.96 (*p* = 0.7)	-7.5 (*p* = 0.48)
**Baseline inhibition**	2.41 (*p* = 0.73)	41.08 (*p* = 0.02)	Not possible to estimate (LL.3 model applied)
**Maximum inhibition** ^**b**^	99.8 (*p* < 0.0001)	99.74 (*p* < 0.0001)	99.98 (*p* < 0.0001)
**ED**_**50**_^**c**^	2.88 µM (*p* < 0.00001, 95% CI, 2.29–3.46 µM)	2.02 µM (*p* < 0.0001, 95% CI, 1.33–2.72 µM)	1.07 µM (*p* < 0.0001, 95% CI, 0.82–1.33 µM)
**ED**_**90**_	4.56 µM (95% CI, 3.36–5.77 µM)	2.93 µM (95% CI, -3.41–9.28 µM)	1.44 µM (95% CI, -0.10–2.99 µM)

a. No significant slope for VGI and VGII reflect high variability between the replicates.

b. *p* values support strong anti-cryptococcal activity.

c. Values below 3 µM for the tested strains indicates that CPTH2 is a potent inhibitor, with significant p values.

d. Modelling for VGI R265 was fitted to a three-parameter log-logistic model (LL.3).

Pharmacodynamic modelling revealed distinct sensitivity profiles among the strains (Table 2, **Figures S3a** and **S3b**). For VGII R265, a three-parameter log-logistic model (LL.3) best fit the data, yielding the lowest ED_50_ (1.07 µM, *p* < 0.0001) and a significant maximum inhibition of 99.98% (*p* < 0.0001). VNI H99_2 and VGI WM1243 also showed significant maximum inhibition (99.8% and 99.74%, respectively; *p* < 0.0001 for both). Among them, VNI H99_2 was the least sensitive, as reflected by its higher ED_50_ (2.88 µM), consistent with its reduced susceptibility in the disc-diffusion assay (17.67 mm; Fig. 1B; Table 1).

Fungicidal activity, assessed by modelling log_10_ CFU reductions using an LL.4 model, further supported CPTH2’s anti-cryptococcal potential. VNI H99_2 exhibited the most robust fungicidal response, with a maximum reduction of 3.60 log_10_ CFU and an ED_50_ of 8.38 µM (*p* < 0.0001) (**Figures S3a** and **S3c**,** Table S1**). VGI WM1243 and VGII R265 displayed lower ED_50_ values (2.72 µM and 1.28 µM, respectively), though accompanied by wider CI and non-significant slope parameters, indicating more variable responses in these two strains. Despite this, all the strains achieved substantial CFU reductions, providing proof of principle that CPTH2 exerts fungicidal activity against *Cryptococcus* under defined in vitro conditions (Fig. 1D).

### Baseline DNA content exhibits strain-specific variation and associates with FLZ susceptibility

**Fig. 2:**
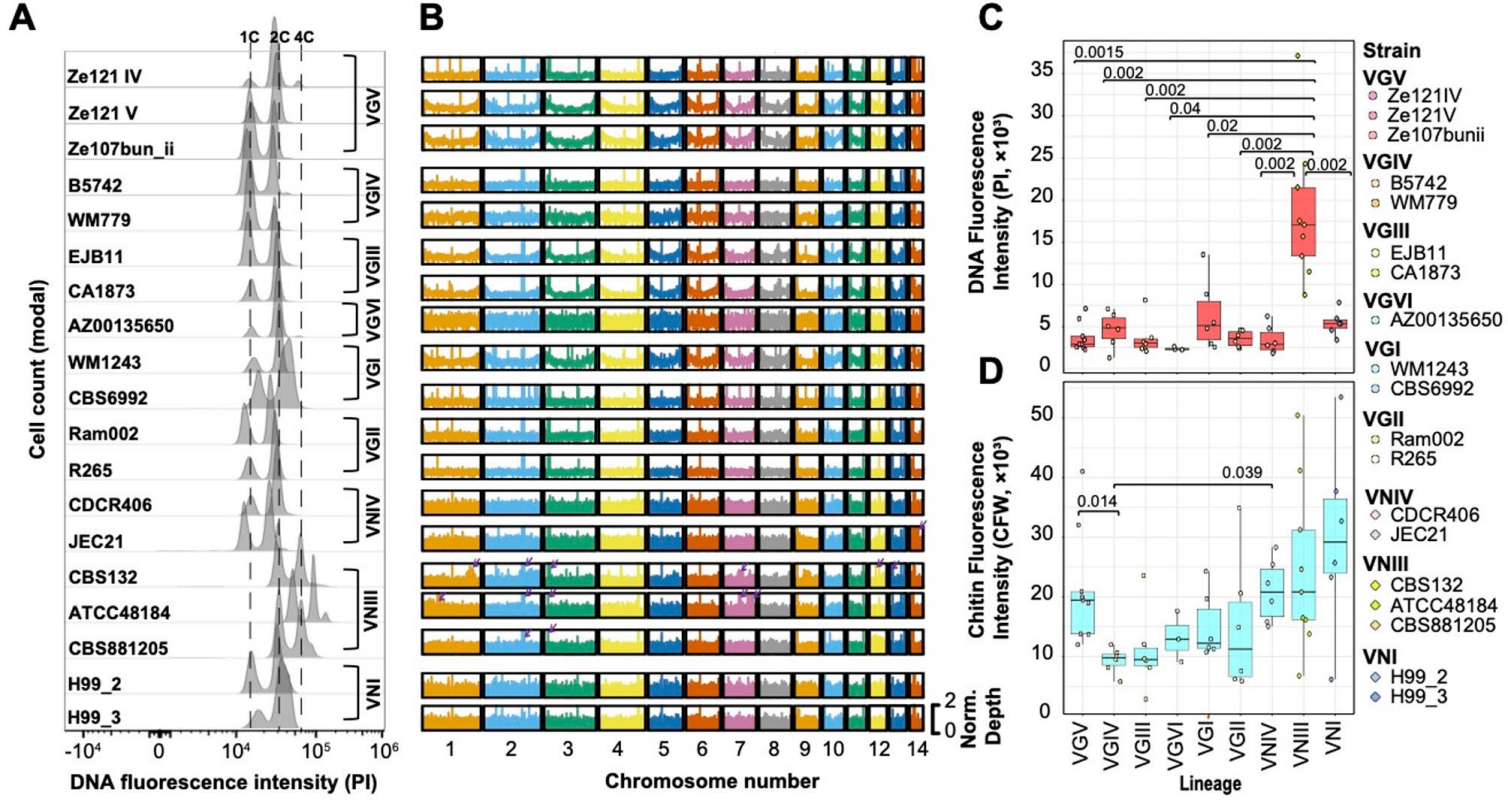
Inter- and intra-lineage variation in DNA and chitin content among *Cryptococcus* lineages. **(A)** Flow cytometry histograms showing DNA content in cells grown in RPMI without antifungal treatment. Cells were stained with propidium iodide (PI) and peaks correspond to 1 C, 2 C, and 4 C DNA content, as marked by dashed lines. Ploidy references were defined using VNI H99_2 (haploid) and VNIII CBS132 (diploid). Lineages are indicated to the right of the histograms. **(B)** Normalised read depth plotted across 1,000 bp non-overlapping windows based on alignments to the *C. gattii* R265 reference genome for *C. gattii* isolates, and the H99 reference genome for *C. neoformans* isolates. Read depth was normalised to the total number of aligned reads per strain. Variations in coverage reflect differences in chromosomal copy number and are indicative of potential aneuploidy. These patterns are consistent with DNA content measured by flow cytometry, particularly within the VNIII lineage. Purple arrows denote regions with elevated read depth, suggesting increased copy number or aneuploidy. (**C**) Box plots of DNA content and (**D**) chitin content by lineage. DNA and chitin levels were quantified by co-staining the cells with PI and calcofluor white (CFW), respectively. Fluorescence intensity (y-axis) reflects values from three independent biological replicates. Each point represents an individual strain and an individual replicate. Inter-lineage comparisons were assessed using the Wilcoxon rank-sum exact test, while intra-lineage variation was evaluated using Tukey’s multiple comparisons of means. Statistically significant intra-lineage variation in DNA content was observed only within the VNIII lineage, suggesting notable ploidy differences (pairwise Wilcoxon tests, FDR-adjusted *q* < 0.05). Inter-lineage differences in DNA content were also statistically significant, particularly between VNIII and other lineages. For chitin content, no statistically significant intra-lineage differences were detected. However, inter-lineage differences were identified with VGIV lineage, showing lower chitin levels compared to VGV and VNIV. Notably, VGIV exhibited the lowest overall chitin content among all lineages. Strains are ordered based on their phylogenetic relationships for consistency and comparative clarity.

While evaluating antifungals susceptibility across *Cryptococcus* strains, the notable variability prompts us to investigate whether basal DNA content could correlate with antifungal susceptibility. Using flow cytometry, we quantified DNA content and found substantial variation across and within *Cryptococcus* lineages. As expected, the highest DNA content was detected in hybrid VNIII isolates (Fig. 2A and B, and 2C). VNIII strains showed significantly higher DNA content than all other lineages (*p* < 0.05), while no significant differences were observed among non-hybrid groups. Notably, variation was evident within VNIII: while the CBS132 and CBS881205 strains had comparable DNA levels, ATCC48184 exhibited significantly higher DNA content (*p* ≤ 0.0005) (Fig. 2C). ATCC48184 also showed reduced susceptibility to 5-FC, FLZ, and CPTH2 (by disc diffusion), suggesting that additional genomic variation may influence antifungal responses.

No consistent intra- or inter-lineage differences in DNA content were detected for the other lineages (VGII, VGIII, VGIV, VGVI, VNI, and VNIV).

To assess whether basal DNA content is associated with antifungal susceptibility, we performed non-parametric correlation analyses. For FLZ, a significant negative correlation was detected between baseline DNA content and growth inhibition (Spearman ρ = -0.32, *p* = 0.012), indicating that strains with higher DNA content tended to exhibit decreased susceptibility to FLZ (**Figure S4a**). To specifically address whether the VNIII strains might disproportionately influence this correlation (higher DNA content of all evaluated lineages), we excluded all three strains. This produced a nearly identical result (Spearman ρ = -0.351, *p* = 0.013) (**Figure S4b**). These findings suggest that DNA content variation (potentially reflecting differences in ploidy or aneuploidy) may influence FLZ response in *Cryptococcus*. However, given the multifactorial nature of antifungal resistance, DNA content alone is unlikely to fully explain the observed susceptibility variation. The association observed provide a proof of principle that genomic plasticity may contribute to antifungal susceptibility, however, validation in larger set of clinical strains and other fungal pathogens, additionally to integration with whole-genome sequencing to identify specific chromosomal alterations will be essential to establish clinical utility.

### FLZ and CPTH2 induce DNA content increase primarily in antifungal susceptible *Cryptococcus* strains

**Fig. 3:**
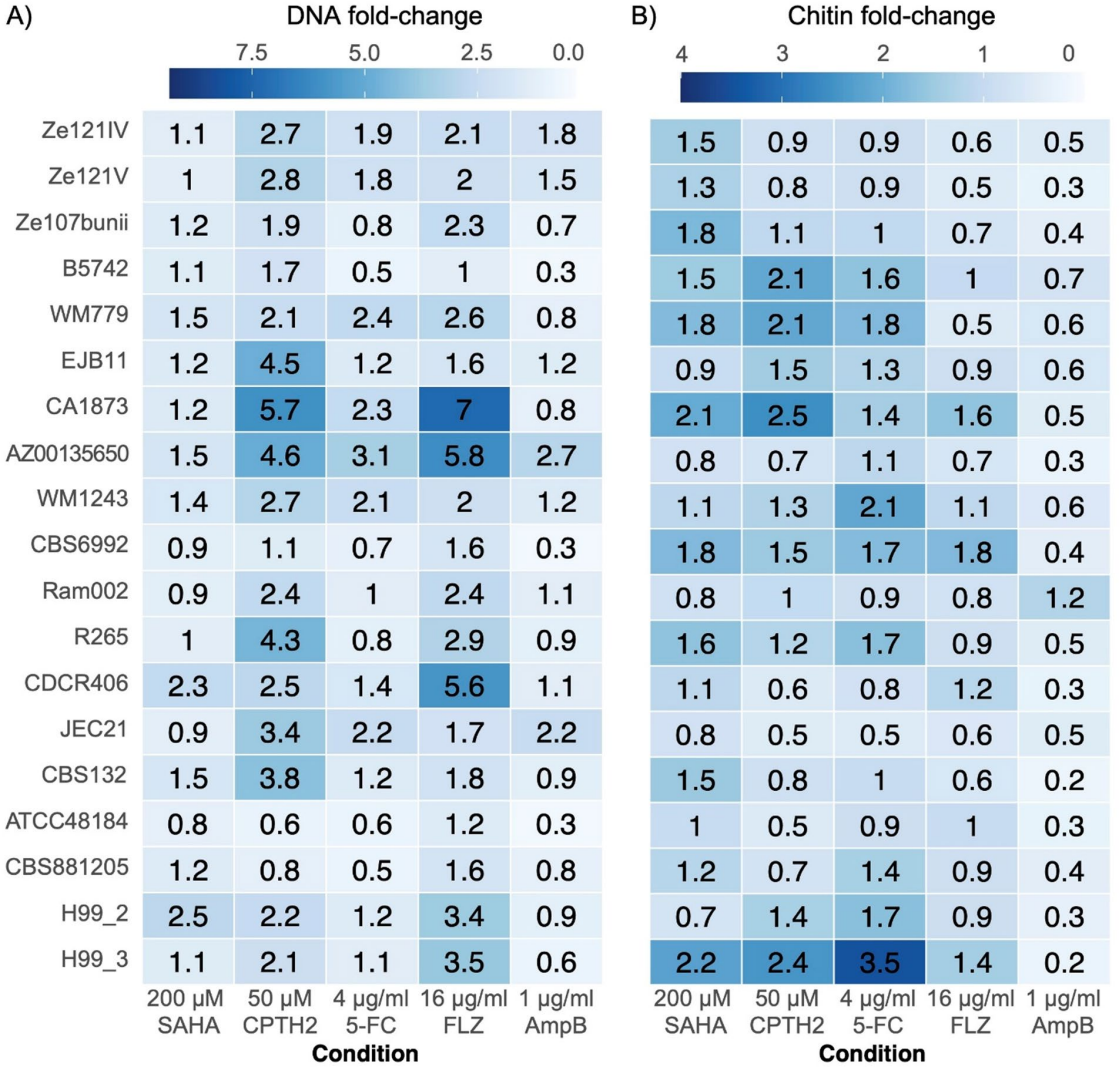
Fold-change in DNA and chitin content following antifungal treatment in *Cryptococcus* strains. Heatmaps showing the fold change in (**A**) DNA and (**B**) chitin content for each strain after treatment with antifungal compounds. Columns represent the compound used to treat the cells, and rows correspond to individual strains, which are clustered according to their phylogenetic relationships. Fold changes are calculated relative to the untreated control for each corresponding strain. Color intensity reflects the magnitude of change (dark blue = greater increase, lighter blue = smaller change or reduction), with a scale bar indicating the range. Numerical values are indicated inside each cell of the heatmap and represent the mean of three independent biological replicates (± SD).

After identifying a correlation between higher basal DNA content and reduced FLZ susceptibility, we next asked whether exposure to antifungal agents could actively alter DNA content in *Cryptococcus*. Such changes could reflect stress-induced genomic responses or adaptive mechanisms contributing to drug tolerance. Notably, FLZ and CPTH2 both induced significant increases in DNA content compared to untreated and DMSO controls (FLZ vs. control: 95% CI = 0.87–2.58, *p* = 0.0000001, CPTH2 vs. control: 95% CI = 0.89–2.59, *p* = 0.0000001). In contrast, DMSO treatment showed no significant difference from the untreated control (95% CI = -0.57–1.13, *p* = 0.96). FLZ had the strongest effect, inducing a 7-fold increase in VGIII strain CA1873 and 5.8-fold in VGVI AZ00135650, followed by CPTH2 (5.7-fold and 4.6-fold in the same strains, respectively) (Fig. 3A). In contrast, no significant DNA content changes were observed with SAHA, 5-FC or AmpB (*p* ≥ 0.69), suggesting that DNA content induction is not a general stress response, but may be specific to FLZ and CPTH2 modes of action.

We compared DNA induction levels across strains with differing susceptibility to FLZ. Strains exhibiting increased FLZ susceptibility (VGVI AZ00135650 and VNIII CBS132) showed markedly greater DNA induction than strains with decreased susceptibility (e.g., VGI CBS6992 and VGIV B5742) (Fig. 3A; Table 1). VGVI AZ00135650 showed a 5.8-fold increase in DNA content under FLZ, while VGI CBS6992 showed only a 1.6-fold increase (*p* = 0.011). Similarly, VNIII ATCC48184 showing decreased susceptibility to FLZ exhibited significantly lower DNA induction (1.2-fold) compared to the susceptible VGVI AZ00135650 (*p* = 0.003).

The VGIII CA1873 strain showed high FLZ inhibition values (94.3% in microdilution and 45.3 mm inhibition zone), despite being classified as indeterminate by both methods. This phenotype is consistent with FLZ susceptibility. Notably, CA1873 also exhibited the highest DNA induction among all tested strains (7-fold increase, *p* = 0.007 vs. VNIII ATCC48184 and *p* = 0.023 vs. VGI CBS6992). This supports the observed trend that higher DNA induction correlates with greater FLZ susceptibility, even when strains do not meet statistical thresholds for formal susceptibility classification (Table 1).

These findings suggest that DNA content increases following FLZ and CPTH2 challenges may reflect a stress response to antifungal pressure, particularly in strains lacking pre-existing resistance mechanisms. Supporting this interpretation, no significant DNA changes were observed in *Cryptococcus* treated with non-FLZ agents (vehicle control DMSO, SAHA, 5-FC and AmpB). Taken together, these observations support a model in which DNA content changes under FLZ and CPTH2 reflect a stress-induced genomic response rather than a resistance mechanism.

### Chitin remodelling varies with antifungal treatment and reflects lineage- and strain-specific stress responses

To explore whether antifungal treatment alters fungal cell wall architecture, we measured chitin content using Calcofluor White (CFW) staining following exposure to various compounds (Fig. 3B). In control conditions, intra-lineage variation in chitin levels was minimal, but significant inter-lineage differences were detected. For example, VGIV strains exhibited among the lowest baseline chitin levels despite showing reduced susceptibility to multiple antifungals (*p* = 0.014 vs. VGV, *p* = 0.039 vs. VNIV; Figs. 1 and 2C), indicating that baseline chitin abundance alone does not predict resistance. Across treatments, modest and variable increases in chitin content were observed following exposure to FLZ, CPTH2, SAHA, and 5-FC, though these changes did not reach statistical significance (FLZ vs. control: 95% CI = -0.48–0.34, *p* = 0.99, CPTH2 vs. control: 95% CI = -0.16–0.65, *p* = 0.55, SAHA vs. control: 95% CI = -0.06–0.75, *p* = 0.17, 5-FC vs. control: 95% CI = -0.03–0.78, *p* = 0.09). In contrast, AmpB significantly reduced chitin levels compared to untreated controls (95% CI = 0.10–0.92, *p* = 0.0043). DMSO treatment showed no significant difference from the untreated control (95% CI = -0.14–0.67, *p* = 0.45).

At the strain level, chitin responses were highly heterogeneous. VNI strains exhibited the strongest chitin induction overall, particularly under 5-FC exposure. Notably, VNI H99_3, a strain with decreased susceptibility to 5-FC, displayed the strongest chitin increase (3.5-fold), and VNI H99_2 also showed elevated chitin levels (1.7-fold) alongside decreased 5-FC susceptibility. In contrast, VNIV strains JEC21, CDCR406, both with high inhibition values to 5-FC (> 91%) exhibited significantly lower chitin induction (0.5–0.8-fold) compared to H99_3 (*p* = 0.03). Although the VNIV strains were classified as indeterminate in susceptibility, their low chitin induction aligns with their higher inhibition levels. Additional strains with decreased 5-FC susceptibility, including VGII R265 and VGIV B5742 and WM779, also exhibited increased chitin induction (1.6–1.8-fold), though these differences were not statistically significant. Importantly, no consistent correlation between chitin induction and antifungal susceptibility was observed across all lineages. Together, these results suggest that chitin remodelling may represent a variable, strain-specific response to antifungal stress rather than a universal or direct resistance mechanism.

### Chitin remodelling as a major driver of phenotypic variation in antifungal stress responses

Chitin fold-change emerged as the strongest contributor to the first principal component (PC1), which explained 24.1% of the total variance (**Table S2**). This was unexpected, as univariate comparisons of chitin content under different treatments (e.g., FLZ, CPTH2, 5-FC and SAHA) had not reached statistical significance. However, the PCA revealed that variation in chitin levels becomes highly informative when considered alongside other phenotypic traits, highlighting its role as a key axis of antifungal stress response rather than an only predictor of resistance.

**Fig. 4:**
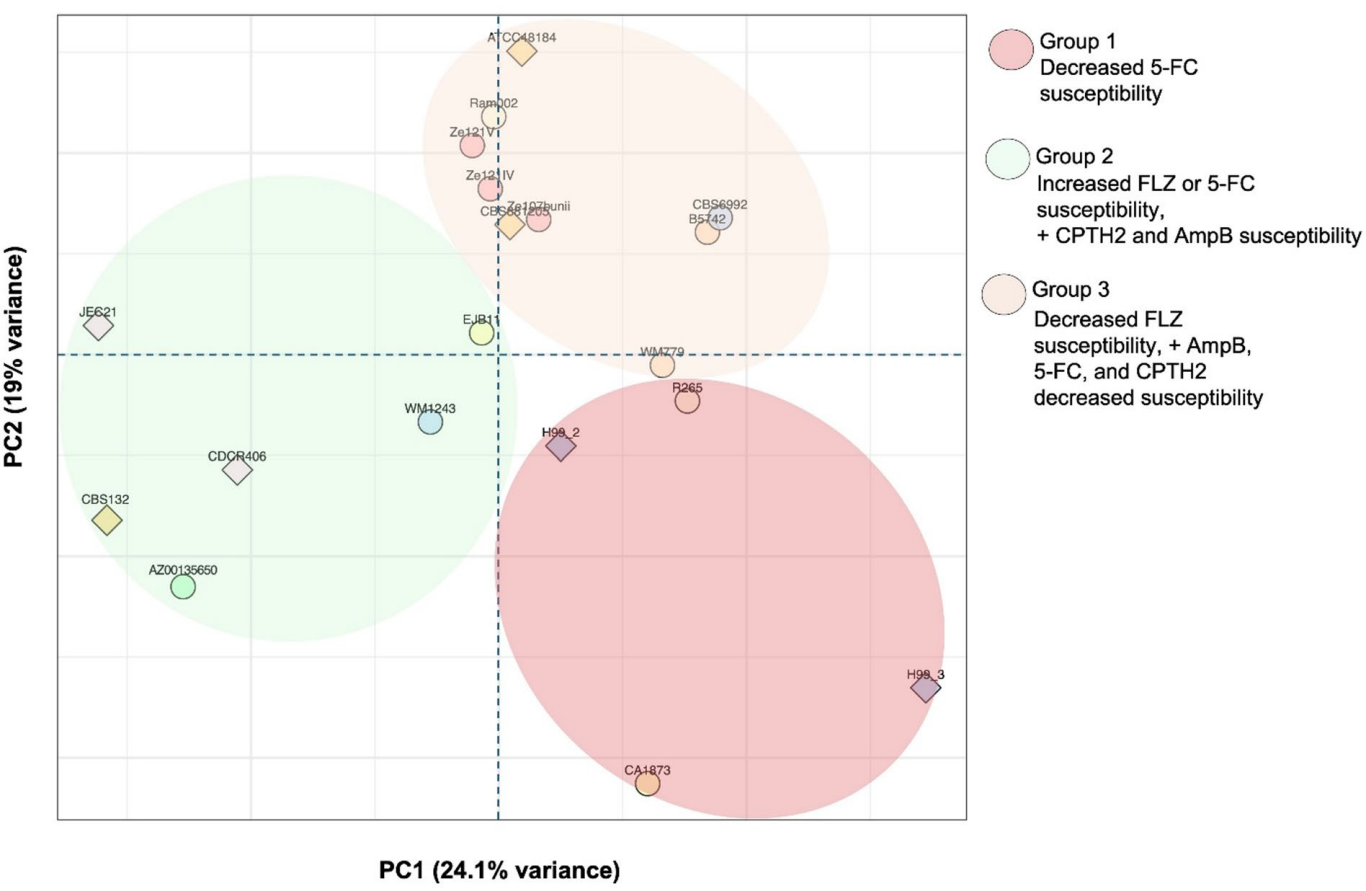
Phenotypic clustering of *Cryptococcus* strains based on principal component analysis (PCA). PCA was performed using the phenotypic traits evaluated in this study, with the first two Principal Components (PC1 and PC2) shown. Strains are colored by unsupervised phenotypic groupings identified through clustering. Pairwise PERMANOVA using Euclidean distance revealed significant group differences between groups (Bonferroni adjusted p values): Group 1 vs. Group 3: R^2^ = 0.431, *p* = 0.006; Group 1 vs. Group 2: R^2^ = 0.425, *p* = 0.024; Group 2 vs. Group 3: R^2^ = 0.5, *p* = 0.003. Cluster stability was further evaluated via bootstrapping (B = 100), resulting in mean Jaccard similarity scores of 0.79, 0.83 and 0.62 for clusters 1, 2 and 3, respectively. Clusters 1 and 2 were considered stable (Jaccard > 0.75), while cluster 3 was less robust. These findings support biologically meaningful separation among the groups.

Based on PCA score distribution and their alignment with phenotypic variables, the strains were grouped into three biologically meaningful clusters (**Table S3**). The clusters showed a strong and statistically significant association with phenotypic groups, as demonstrated by PERMANOVA test on the first three PCs (R^2^ = 0.52, *p* = 0.001). Pairwise PERMANOVA comparisons further confirmed significant differences between all group pairs after Bonferroni correction (*p* = 0.006 for Group 1 vs. Group 3; *p* = 0.003 for Group 2 vs. Group 3; *p* = 0.024 for Group 1 vs. Group 2) (Fig. 4). Bootstrap analysis of group centroids in PCA space showed consistent separation among phenotypic groups (**Figure S5a**). The 95% CI for the average PC1, PC2 and PC3 scores of each group did not overlap substantially (**Table S4**), supporting robust clustering.

Consistent with these multivariate patterns, VGIV strains, previously noted for their low baseline chitin content, consistently showed among the highest levels of chitin induction following antifungal treatment (Fig. 3B). This suggests a potential lineage-specific adaptation in which inducible chitin remodelling may partially compensate for inherently low basal chitin levels under stress conditions. Overall, these analyses indicate that while chitin remodelling does not uniformly correlate with resistance, it constitutes a major phenotypic dimension shaping strain-specific antifungal stress responses in *Cryptococcus.*

## Discussion

Cryptococcosis remains a life-threatening fungal infection, particularly in resource limited regions where access to effective antifungal therapy is constrained. One of the most pressing clinical challenges is the intrinsic resistance of *Cryptococcus* to echinocandins, one of the safest antifungal classes^1,19,21^. This limitation is further compounded by rising rates of FLZ resistance, frequent relapse, and therapeutic failure^35^. A critical component of this problem is our limited understanding of the molecular mechanisms that drive antifungal resistance in *Cryptococcus*. Without a clear understanding of how resistance emerges and evolves, treatment strategies often lead to suboptimal outcomes (e.g. relapse, treatment failure). In this context, there is an urgent need for novel antifungal agents with broad spectrum activity and low host toxicity, in combination with new insights into resistance pathways that could inform more targeted and effective therapeutic interventions.

Here, we investigated CPTH2 as a promising anti-cryptococcal candidate and demonstrated its superior in vitro activity compared to commonly used agents (5-FC and FLZ). CPTH2 inhibited fungal growth and demonstrated fungicidal activity in vitro. This offers a proof of principle for its therapeutic potential. CPTH2 exhibited a potent and broad-spectrum activity across all tested lineages, with relative growth values ranging from 1.23% to 5.15% (at 50 µM), a concentration lower than that required to inhibit the human ortholog^30^. This is striking, considering the high host toxicity of current antifungals^12,13,36^. The pharmacodynamic modelling revealed near complete growth inhibition at higher concentrations. Additionally, its fungicidal activity, demonstrated by significant reduction in CFUs (3.60 log_10_ CFU reduction in VNI H99_2, and 3.95 for VGI WM1243) provides a strong foundation for further drug development. These effects stand in contrast to the variable efficacy observed with standard antifungals (relative growth under FLZ and 5-FC ranged from 2.41% to 56.8% and 3.96% to 57.7%, respectively).

Notably, CPTH2’s spectrum is not limited to *Cryptococcus*. It has also shown efficacy against *C. auris* and members of the CTG clade (*C. albicans*), broadening its potential clinical utility^30,31^. Given the significant host toxicity associated with the only fungicidal agent in the current arsenal (AmpB), our results along with other recent reports on CPTH2 underscore its value as a lead compound for future antifungal drug development.

Despite the promising results and the fungicidal potential demonstrated by CPTH2, several methodological considerations must be considered when interpreting these findings. A weak correlation was observed between the disc-diffusion and the microdilution assays, suggesting potential limitations of the disc-diffusion method, possibly related to CPTH2’s solubility or diffusion properties in the agar media. These discrepancies underscore the importance of employing complementary methodologies when assessing compounds with less characterised pharmacodynamics. Notably, the disc-diffusion method identified VNI H99_2 as a strain with one of the smallest inhibition zones, falling close to the threshold for decreased susceptibility. Pharmacodynamic modelling supported this observation, showing that H99_2 required the highest CPTH2 concentration to achieve growth inhibition (ED_50_ 2.88 µM). This reinforces the value of employing diverse testing approaches and highlights the role of strain specific genetic variability in shaping antifungal susceptibility^37,38^. Additionally, these observations warrant further investigation to assess CPTH2’s efficacy across larger clinical isolate panels and to determine whether the observed strain-specific differences reflect intrinsic genetic variation or represent early markers of potential resistance mechanisms. Together, these findings support the need for precision driven therapeutic strategies to reduce treatment failure and resistance development in *Cryptococcus* infections.

A central aim of this study was to characterise how *Cryptococcus* strains respond to antifungal stress across multiple phenotypic dimensions. Fungal pathogens can respond to antifungal exposure through several, often partially independent, adaptative pathways. Among these, genomic plasticity (reflected by changes in DNA content) and cell wall remodelling (reflected by changes in chitin content) represent responses acting along distinct biological axes: one addressing transcriptional needs and the other maintaining structural integrity. Building on previous evidence linking genomic plasticity to antifungal stress responses^23,25,26,39^, we explored how *Cryptococcus* dynamically modulates its DNA content in response to antifungal exposure. Using flow cytometry as a proxy for genome content, we quantified global changes in cellular DNA levels across strains and treatment conditions. Our analyses revealed two key patterns: (*i*) we detected a significant inverse relationship between baseline DNA content and FLZ susceptibility, particularly evident in certain strains within the VNIII and VGI lineages. This finding likely reflects the role of aneuploidy in modulating the expression of resistance associated genes (e.g. through duplication of chromosome 1)^39^; and (*ii*) FLZ and CPTH2 exposure in susceptible strains like VGVI AZ00135650 triggered striking DNA increase (> 4-fold for both conditions). Notably, these DNA content increases occurred in strains exhibiting increased susceptibility rather than decreased susceptibility, indicating that DNA amplification does not immediately confer protection but rather represents a transient, stress induced response.

The fate of these DNA content changes following removal of antifungal pressure remains unclear. Because our study focused on endpoint measurements after acute drug exposure, we were unable to determine whether the observed increases represent stable chromosomal duplications or transient genome instability. Whether antifungal-induced genomic instability serves as an early step in the evolution of stable resistance or instead reflects cellular dysfunction under stress remains an important question for future investigation. Microevolution experiments combined with longitudinal monitoring of DNA content could help determine whether such populations are predisposed to acquiring stable resistance mutations over time. Finally, we highlight the utility of flow cytometry to determine DNA content as a rapid, useful biomarker of antifungal stress in *Cryptococcus*, though its predictive power for resistance remains limited. Further whole genome sequencing or chromosome specific approaches are required to characterise the genomic basis of observed DNA content changes.

We found that chitin content only had minor changes in response to antifungals. However, multivariate PCA analysis revealed its relevance under treatment-induced stress, emphasizing the complexity of the cryptococcal response landscape. Furthermore, chitin remodelling emerged as a principal driver of phenotypic variation, contributing significantly to PC1 (24.1% of variance) and allowing strains to cluster into three distinct phenotypic groups (Fig. 4, **Table S3**). This finding was unexpected given the modest fold-changes observed in univariate analyses and highlights the value of integrative approaches in uncovering subtle, yet functionally relevant phenotypic differences that distinguish resistant and susceptible strains. Thus, chitin remodelling appeared particularly relevant in the response to 5-FC. VGIV strains, which exhibited the lowest baseline chitin levels, strongly induced chitin (> 1.6-fold) following 5-FC exposure, suggesting a compensatory mechanism to reinforce cell wall integrity under stress. Similarly, VNI strains, already exhibiting the highest basal chitin levels, further increased chitin (> 1.7-fold) and showed decreased susceptibility to 5-FC. In contrast, more susceptible VNIV strains exhibited minimal chitin changes (< 0.8-fold). These patterns support the idea that chitin remodelling acts as a flexible and inducible defence strategy, with its importance varying across strains.

Our findings are consistent with growing evidence that implicates the cell wall as a key player in 5-FC response. In *C. glabrata*, exposure to 5-FC induces rapid cell wall damage followed by structural reinforcement, indicating an active remodelling process in response to the drug. Similarly, chemogenomic studies in *Saccharomyces cerevisiae* identified cell wall remodelling as one of the key contributors to 5-FC resistance^40^. These mechanisms may act independently or in synergy with mutations in resistance genes such as *FCY2* and *FUR1*, or with altered nucleotide flux as seen in VGII strains carrying mutations in *UXS1*, a gene involved in capsule and nucleotide metabolism^41^. Together, our findings underscore the concept that 5-FC resistance is multifactorial, extending beyond classical targets (e.g. DNA-related pathways), but also including broader, strain-specific adaptive responses such as cell wall remodelling.

CPTH2 demonstrates potent, broad-spectrum fungicidal activity against all major *Cryptococcus* lineages tested, positioning it as a promising antifungal candidate, particularly in regions with limited access to liposomal AmpB and 5-FC. By targeting Gcn5^30,31^, CPTH2 may circumvent traditional resistance mechanisms while simultaneously addressing a recently discovered resistance pathway. Gouveia-Eufrasio et al.. demonstrated that *C. gattii* employs *GCN5* to enzymatically inactivate FLZ through acetylation, converting it to *O*-acetyl-fluconazole, a metabolite unable to bind its target lanosterol 14α-demethylase. Critically, CPTH2 treatment abolished this acetylation, restoring FLZ efficacy^42^. While our study focused on growth inhibition and cellular stress responses (DNA and chitin dynamics), the mechanism of action of CPTH2 in *Cryptococcus* warrants further investigation. In *C. auris*, inhibition or deletion of *GCN5* modulates the expression of genes involved in ergosterol biosynthesis (*ERG11*,* ERG1*,* ERG3* and *ERG25*), efflux pump activity (*MDR1*,* CDR1*, and *AFR1-3*), and cell wall integrity (*CAS5*)^30,43^. If CPTH2 acts through similar mechanisms in *Cryptococcus*, we would predict pleiotropic effects: *i*) impaired ergosterol synthesis; *ii*) reduced efflux pump expression; and *iii*) compromised cell wall integrity. Beyond ergosterol-associated effects, *GCN5* in *Cryptococcus* regulates key virulence determinants including capsule formation, melanin production and thermotolerance^44,45^. Future studies should therefore address CPTH2’s impact not only on ergosterol content, efflux pump expression, and cell wall composition, but also on virulence factor production through integrated approaches combining transcriptomics, biochemical profiling and in vivo virulence assays.

To determine the potential therapeutic benefit of CPTH2, in vivo validation of its efficacy, safety and pharmacological properties is needed. While we did not directly assess cytotoxicity in this study, published data suggest that CPTH2 has a favourable safety profile^30,31^. Zhang et al. reported no notable acute host toxicity in murine candidiasis models. Similarly, Tscherner et al. observed no significant effect on HEK293 cell viability^30,31^. This selectivity likely reflects structural differences between fungal and mammalian *GCN5* orthologs, though the therapeutic window for cryptococcal infections requires formal characterisation through: *i*) pharmacokinetics and blood-brain barrier penetration studies, these are critical parameters for treating CM; *ii*) efficacy assessments in murine models of pulmonary cryptococcosis and meningoencephalitis; *iii*) toxicology studies establishing maximum tolerated doses and evaluating potential chronic organ toxicity (hepatic, renal, hematologic); and *iv*) validation that the EC_50_ concentrations identified in our study are achievable and safe in vivo. Given the strain-specific variation in CPTH2 susceptibility observed here, pharmacodynamic modelling across diverse clinical isolates will be essential to guide dosing strategies. Investigating CPTH2 in combination with existing antifungals may offer synergistic effects, thereby improving therapeutic outcomes while potentially reducing drug-related toxicity, an approach that has shown promise in *C. auris*^30^. Such strategies are especially relevant for addressing the high burden and mortality of cryptococcosis in LMICs.

The integration of multi-parametric phenotyping (combining growth inhibition with DNA and chitin content profiling), may provide a more comprehensive framework for antifungal surveillance and resistance monitoring. The association between DNA content and FLZ susceptibility (**Figure S4**), though requiring validation in larger cohorts, highlights the potential value of integrating genomic surveillance with phenotypic profiling to predict resistance risk and guide therapy. Similarly, the emergence of chitin remodelling as a principal component in phenotypic clustering suggests its potential utility as a biomarker for antifungal stress response, especially in lineages expressing low chitin levels like VGIV.

In summary, our study (*i)* highlights CPTH2’s potential as a novel broad anti-cryptococcal agent, and (*ii*) reveals strain-specific variation in DNA and chitin responses to antifungal stress that highlight the multifaceted nature of cellular adaptation. We demonstrated that *Cryptococcus* strains employ diverse, often independent adaptative strategies encompassing both genomic plasticity and cell wall remodelling. However, several limitations moderate these conclusions. Our sample size restricts definitive conclusions about lineage-wide patterns, the observed variation may reflect strain-specific adaptations rather than lineage-level traits. Additionally, our measurements captured acute stress responses rather than long term stability of DNA content changes, and in vivo validation remains necessary to assess CPTH2’s therapeutical potential. These findings nonetheless support a multifaceted approach to antifungal development and surveillance, one that integrates phenotypic, genomic and multivariate analyses to deepen our understanding on drug responses and guide the design of more effective and accessible therapies for cryptococcosis. Future work incorporating larger strain panels, temporal analyses, and molecular characterisation of stress response mechanisms will be essential to translate these findings into clinically actionable resistance biomarkers and therapeutic strategies.

## Materials and methods

### Strains, media, chemicals and growth conditions

Isolates belonging to the different phylogenetic lineages of the genus *Cryptococcus* were collected from three different laboratories: Professor Robin May, University of Birmingham, UK, Professor Matthew Fisher, Imperial College, London, UK, and Professor David Engelthaler, Translational Genomics Research Institute, Flagstaff, Arizona. It is a compendium of clinical, veterinary and environmental samples (**Table S5**). Each collected isolate was recovered from a stock in -80 °C or liquid nitrogen. Cells were thawed and streaked in YPD agar (1% yeast extract, 2% bacto-peptone, 2% glucose, 2% bacto-agar) (Sigma Y1375), 30 °C for 48 h to produce single colonies. One single colony was inoculated into YPD liquid + 2% glucose and incubated overnight at 30 °C, in a rotary shaker at 200 rpm. The strains were preserved in 20% glycerol and frozen stocks were recovered for each experiment in YPD agar. RPMI-1640 medium was purchased from Merck (R6504). CPTH2 (Biorbyt™ catalogue number orb1299016), SAHA (Sigma SML0061), fluconazole (Acros organics™ catalogue 455480010), amphotericin B (Sigma A4888), stock solutions were prepared in dimethyl sulfoxide (DMSO). 5-flucytosine (Sigma F0175000) stock solution was prepared in sterile water. Strains used in this study are listed in **Table S5**.

### Genomic DNA isolation for sequencing

Cells were grown in YPD + 2% glucose agar for 48 h to obtain single colonies. One single colony was inoculated in YPD + 2% glucose for 16 h at 30 °C, with shaking at 200 rpm. DNA was isolated using a combination of phenol chloroform-isoamyl-alcohol method and the Qiagen DNA mini kit (QIAmp DNA mini kit, 51304)^46^. Briefly, cells were resuspended in 200 µl of Tris EDTA buffer and 200 µl of phenol, followed by vortexing (20 s vortex: 20 s ice, repeated for 3 cycles). The samples were snap-frozen in liquid nitrogen, then heated to 75 °C for 10 min. Next, 200 µl of chloroform: isoamylalcohol (24:1) was added and the lysate was centrifuged to separate the aqueous phase. A second chloroform: isoamylalcohol (24:1) wash was performed to remove residual phenol. The aqueous phase was precipitated with 2 volumes of absolute ethanol and incubated at -80 °C for at least 2 h. The DNA was resuspended in 400 µl of 10 mM Tris HCl pH 8.0 and treated with RNaseA (100 µg/ml) at 37 °C for 30 min. DNA purification was completed using the Qiagen kit by adding 1.5 volumes of buffer AW1, followed by transfer to the DNeasy mini spin column. The column was centrifuged and washed with buffer AW2, followed by elution with 10 mM Tris HCl pH 8.0.

DNA was quantified using Qubit 4.0 Fluorometer (Invitrogen, USA) and the integrity was checked on agarose gel. Genomic libraries were prepared and sequenced in the Sequencing Facility at Exeter University. A NovaSeq6000 platform was used.

### Genomic and phylogenetic analysis

The Genome Analysis Toolkit (GATK) v.4.1.2.0 was used to call variants^47^. Briefly, raw sequences were pre-processed by mapping reads to the reference genome *C. gattii* R265 using BWA-MEM v.0.7.17^48^. Next, duplicates were marked, and the resulting file was sorted by coordinate order. Intervals were created using a custom bash script allowing parallel analysis of large batches of genomic data. Using the scatter-gather approach, HaplotypeCaller was executed in GVCF mode with the haploid ploidy flag. Variants were imported to GATK 4 GenomicsDB and hard filtered if QualByDepth (QD) < 2.0, FisherStrand (FS) > 60.0, root mean square mapping quality (MQ) < 40.0, genotype quality (GQ) ≥ 50, allele depth (AD) ≥ 0.8, or Coverage (DP) ≤ 10.

To identify aneuploid chromosomes, depth of coverage was calculated for each isolate. Sorted BAM files prepared in the pre-processing phase of SNP calling were passed to Samtools v.1.2^49^ and mpileup files were generated. Read depth was normalized by total alignment depth and plotted against the location in the genome using 10 kb nonoverlapping sliding windows.

To construct species-specific phylogenetic trees, all sites that were either a homozygous reference or SNP in every isolate were identified using ECATools (https://github.com/rhysf/ECATools) and concatenated into a FASTA file. Our midpoint tree included 6967 phylogenetically informative sites. Phylogenetic trees were constructed with RAxML PThreads v.7.7.8^50^ using the general-time reversible model and CAT rate approximation with 1000 bootstrap support.

### *Cryptococcus* drug susceptibility testing

We used the EUCAST broth microdilution method for antifungal susceptibility testing, with the specific aim of evaluating responses to a single concentration across lineages. This design allowed us to simultaneously compare growth inhibition, DNA and chitin content within the same experimental framework, maximising data readout per assay. Because clinical breakpoints are not established for *Cryptococcus*, we relied on tentative EUCAST-based ECVs reported by Cordoba et al. 2016 and Espinel-Ingroff et al. 2023 to guide concentration selection^32,33,51^. For standard antifungals, we chose clinically relevant concentrations commonly used in vitro: 4 µg/ml for 5-FC, 16 µg/ml for FLZ, and 1 µg/ml for AmpB. For the epigenetic inhibitors, no standard concentrations or breakpoint exist, we therefore performed preliminary dose-range assays (50–200 µM) for CPTH2 and for SAHA. CPTH2 showed reproducible antifungal activity even at the lowest concentration tested initially (50 µM), whereas SAHA showed no measurable activity up to 200 µM. Based on these results, we selected 50 µM CPTH2 and 200 µM SAHA as working concentrations for comparative phenotypic profiling. Although SAHA lacked antifungal activity, its inclusion as a HDACi control strengthened our conclusions: only CPTH2 induced DNA content changes and growth inhibition, indicating that these phenotypes reflect antifungal action rather than a generic effect of chromatin modulation.

Growth inhibition was quantified following the EUCAST broth microdilution guidelines^52^, across all recognised lineages of *C. neoformans* and *C. gattii* (two representative strains per lineage, unless indicated). Disc-diffusion assays were performed according to the protocol of Barry et al. (2002) with minor modifications^52,53^. Broth microdilution assays were conducted in RPMI-1640 medium (Merck R6504) supplemented with 2% glucose and buffered with MOPS to pH 7^52^. *Cryptococcus* cells were pre-grown for 16 h in YPD + 2% glucose at 30 °C, 200 rpm. 1 × 10^5^ cells per ml were inoculated in a 96-well plate containing the appropriate antifungals (50, 100 and 200 µM for CPTH2 and SAHA, 4 µg/ml for 5-FC, 16 µg/ml for FLZ and 1 µg/ml for AmpB). Plates were incubated without agitation at 30 °C for 48 h. Each assay included blank wells (medium only), growth controls (cells with DMSO at the final concentration used in the drug wells), and the wells with the compound. Growth was quantified by measuring the absorbance at 530 nm with a Tecan Spark^®^ multimode microplate reader. Relative growth was calculated as:

$$\:Relative\:growth=\frac{Ab{s}_{compound}-Ab{s}_{blank}\:}{Ab{s}_{control}}\times\:100$$

Disc-diffusion assays were carried out using Mueller Hinton agar (Merck 70191) supplemented with 2% glucose and 0.5 µg/ml methylene blue dye. 3 × 10^6^ *Cryptococcus* cells were inoculated in the plate, the inoculum was evenly distributed in the plate and was left to dry for 10 min. The disc was then applied and gently pressed onto the agar. One disc per plate was used. The plates were incubated at 30 °C for 72 h and the inhibition areas were determined (area in which there is no visible growth of the fungus). OxoidTM Blank Antimicrobial Susceptibility Discs (Fisher Scientific™ CT0998B) were loaded with the appropriate concentrations of the compounds to test (320 µg FLZ, 18 µg AmpB, and 175 µg CPTH2). Disc controls loaded with just DMSO were included in the experiments, as well as disc controls not loaded with any drug.

### Drug response modelling for CPTH2

Because CPTH2 showed antifungal activity in vitro, we further aimed at determining the effective concentrations of CPTH2 required to inhibit *Cryptococcus* growth, a two-fold serial dilution of the compound was tested, ranging from 1 to 128 µM. Growth inhibition was assessed using a broth microdilution assay following the EUCAST guidelines. After 48 h of incubation at 30 °C, cells were washed twice with PBS 1X, and appropriate dilutions were seeded in YPD plates containing 2% glucose. Plates were incubated for an additional 48 h at 30 °C to determine the presence of any viable cell by monitoring colony formation. Untreated and DMSO controls were included in all experiments. Three biologically independent experiments were performed. Log_10_ reductions were calculated by comparing CFUs from treated samples to those from untreated controls. This allowed us to quantify the fungicidal activity of CPTH2:

$$\:Log10\:reduction=log10\:(\frac{CF{U}_{control}\:}{CF{U}_{CPTH2}})$$

Dose-response curves were fitted using a four-parameter log-logistic model (LL.4), via the drc package in R^54^, except for the strain VGII R265, where we applied a three-parameter log-logistic model (LL.3), due to non-identifiable baseline inhibition values. From the models, key pharmacodynamic parameters were extracted: slope, minimum and maximum inhibition and the effective doses causing 50% and 90% inhibition (ED_50_ and ED_90_, respectively) (Table 2). To assess fungicidal activity, CFUs were enumerated after drug exposure (Fig. 1D). Log_10_ reductions were modelled using the four-parameter log-logistic model (LL.4) (**Table S1**). Both assays allowed us to distinguish between fungistatic and fungicidal effects of CPTH2.

### *Cryptococcus* cell fixation for DNA and chitin content determination

For analysis of DNA and chitin content, unless otherwise stated, cells were grown on 96-well plates on RPMI + 2% glucose (EUCAST media) for 48 h. Cells were fixed with a mixture of methanol: glacial acetic acid (3:1). Briefly, cells were washed with 50 mM sodium citrate twice. Then, cells were fixed with 150 µl fixation buffer (70% methanol, 20% glacial acetic acid, 10% DMSO, 0.1% triton X-100 and 5mM EDTA)^55,56^, incubated on ice during 15 min. The fixative was then washed with 0.1% triton X-100, and finally the cells were washed once with 50 mM sodium citrate. Subsequently, cells were treated with 200 µg/ml RNaseA, incubated at 37 °C during 2.5 h. After this, RNaseA was washed with 50 mM sodium citrate and cells were resuspended in a 50 mM sodium citrate solution containing 25 µg/ml propidium iodide (PI) and incubated at 37 °C over night (cells were kept in the dark). On the following day, 0.25 µg/ml calcofluor white (CFW) was added and cells were analysed in the flow cytometer.

### Flow cytometry analyses

Flow cytometry assays were performed using an Attune NxT Flow Cytometer^®^ (Thermo Fisher Scientific). To determine DNA and chitin content, samples were kept in the dark until analysis. For CFW, VL1 channel (417 nm excitation, 450/40 nm emission) was used. For PI, YL2 channel was used (561 nm excitation, emission at 620/15 nm). A total of 20,000 events were acquired based on Forward Scatter (FSC) and Side Scatter (SSC). FlowJo™ software was used for data acquisition and analysis. Controls consisted of unstained cells, cells single stained with CFW and single stained with PI. Doublets were filtered out using comparisons of FSC-H vs. FSC-W^55,56^.

DNA content was assessed using the Median Absolute Deviation (MAD), while chitin content was measured via Mean Fluorescence Intensity (MFI). To account for variability between strains and the experimental replicates, fluorescence signals were normalised to the untreated control, with values expressed as fold-changes relative to the control. A value greater than 1 indicates an increase in DNA or chitin content relative to the cells in the control condition (media without compounds). Data were calculated from three independent biological replicates.

### Principal component analysis for the phenotypic data

To assess how all the phenotypic traits we evaluated in this study contribute to antifungal susceptibility profiles, we performed principal component analysis (PCA) incorporating relative growth, zone of inhibition diameter, and fold-changes in DNA and chitin content following antifungal treatment. This multivariate approach allowed us to capture coordinated phenotypic patterns across strains and identify features driving their differential responses (Fig. 4). Multivariate statistical analysis (principal component analysis PCA) was performed using phenotypic data collected from the *Cryptococcus* strains exposed to the antifungal compounds (SAHA, CPTH2, 5-FC, FLZ and AmpB) and to the vehicle control DMSO. The dataset included relative growth measurements, inhibitory zone diameters, and fold-changes in DNA and chitin content, calculated relative to untreated controls. Prior to PCA, all variables were standardised (mean-centred and scaled to unit variance) to account for differences in measurement scale. We used the prcomp function from R, with default settings to perform the PCA. The first three components (PC1, PC2 and PC3) were selected as allowed us to detect phenotypic shifts associated with increased or decreased susceptibility to the antifungals tested. Based on PCA score distribution and alignment with phenotypic profiles, strains were grouped into three biologically meaningful clusters. To evaluate robustness of group separation, bootstrap resampling (1000 iterations) was performed to compute 95% CI for the PCA centroids of each phenotypic group. The statistical significance of the three clusters identified was evaluated using a permutational multivariate analysis of variance (PERMANOVA, adonis2 from the vegan package, 999 permutations) using Euclidean distances applied on the first three principal components.

### Statistical analysis

All statistical analyses were conducted in R (version 2024.12.1 + 563)^57^. Experiments followed a paired design (untreated vs. treated strains) and were performed in three independent biological replicates, each including multiple readouts per sample (growth, inhibition area, DNA and chitin content). Because we tested only a single concentration per drug, we could not assign clinical susceptibility categories (susceptible or resistant). Instead, we compared strains relative to the cohort median. We defined three cohort-based, descriptive categories: decreased, increased, or indeterminate susceptibility. To classify strain phenotypes, for the microdilution method, for each antifungal drug, percent inhibition (100 – relative growth) was standardised across strains using a robust *z*-score: *z =* (x - median)/MAD, where MAD was scaled by 1.4826. Strains were classified as having decreased susceptibility if (*z* ≤ -0.8 or ≤ 25th percentile) and ≤ -5% growth inhibition below cohort median. Strains were classified as having increased susceptibility if (*z* ≥ 0.8, or ≥ 75th percentile) and ≥ + 5% growth inhibition above cohort median. Otherwise, strains were classified as having indeterminate susceptibility. For the disc-diffusion method, strains were classified as having decreased susceptibility if (*z* ≤ -0.7 or ≤ 25th percentile) and ≤ -3 mm inhibition below cohort median. Strains were classified as having increased susceptibility if (*z* ≥ 0.7, or ≥ 75th percentile) and ≥ + 3 mm inhibition above cohort median. Otherwise, strains were classified as having indeterminate susceptibility.

To assess statistically significant differences between strains under different conditions, we used contrast functions from the emmeans package. Pairwise comparisons were adjusted for multiple testing using Tukey’s Honest Significant Difference (HSD) method. The correlation between the microdilution and disc-diffusion assays was evaluated using Pearson’s correlation coefficient. Pharmacodynamic modelling for CPTH2 was performed using the drc package^54^. A dose-response modelling using four-parameter-log-logistic model was employed for VNI H99_2 and VGI WM1243 (LL.4), while for VGII R265, a three-parameter-log-logistic model was used (LL.3). For pairwise comparisons of DNA and chitin content between lineages, the Wilcoxon rank-sum exact test was applied. P-values were adjusted for multiple testing using the Benjamini-Hochberg false discovery rate (FDR) method. Differences within lineages were assessed using one-way ANOVA, followed by Tukey’s Honest Significant Difference (HSD) for post hoc multiple comparisons. Statistical significance was defined as *p* < 0.05.

## Supplementary Information

Below is the link to the electronic supplementary material.

Supplementary Material 1

Supplementary Material 2

Supplementary Material 3

Supplementary Material 4

Supplementary Material 5

Supplementary Material 6

## Data Availability

Sequencing reads are available at the NCBI sequence read archive under accession numbers PRJNA1279626 ( *C. neoformans* strains) and PRJNA1279655 ( *C. gattii* strains).
