# Supplementary figures and images for: Antifungal-induced DNA dynamics and chitin remodelling across Cryptococcus spp. and the novel broad-spectrum anti-cryptococcal candidate CPTH2
Source: Sci Rep. 2026 May 19;16:22622. doi: 10.1038/s41598-026-52566-9 (PMC13381734; doi:10.1038/s41598-026-52566-9)

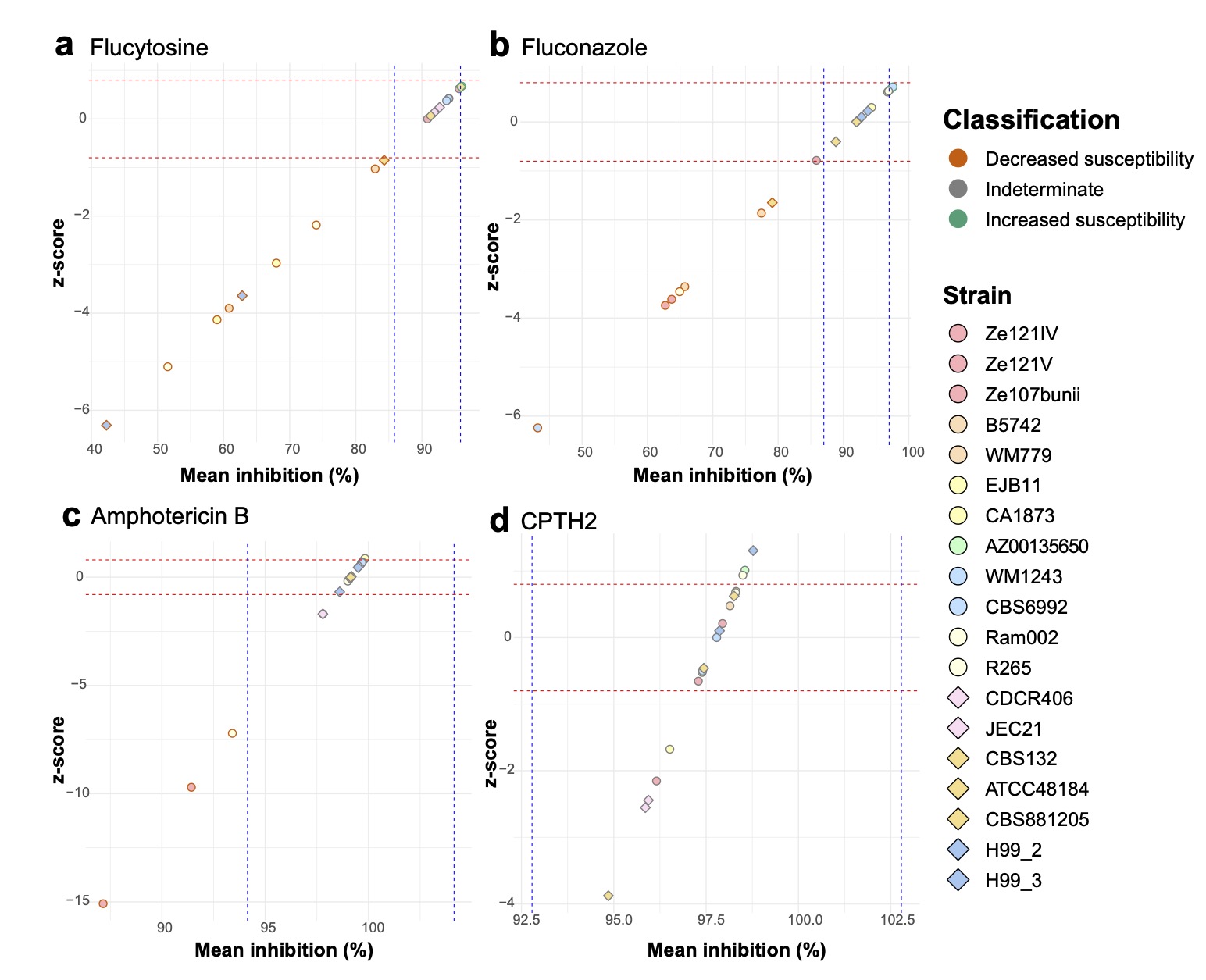

Supplement: Supplementary file 1 — Supplementary Material 1 [file 41598_2026_52566_MOESM1_ESM.jpg]

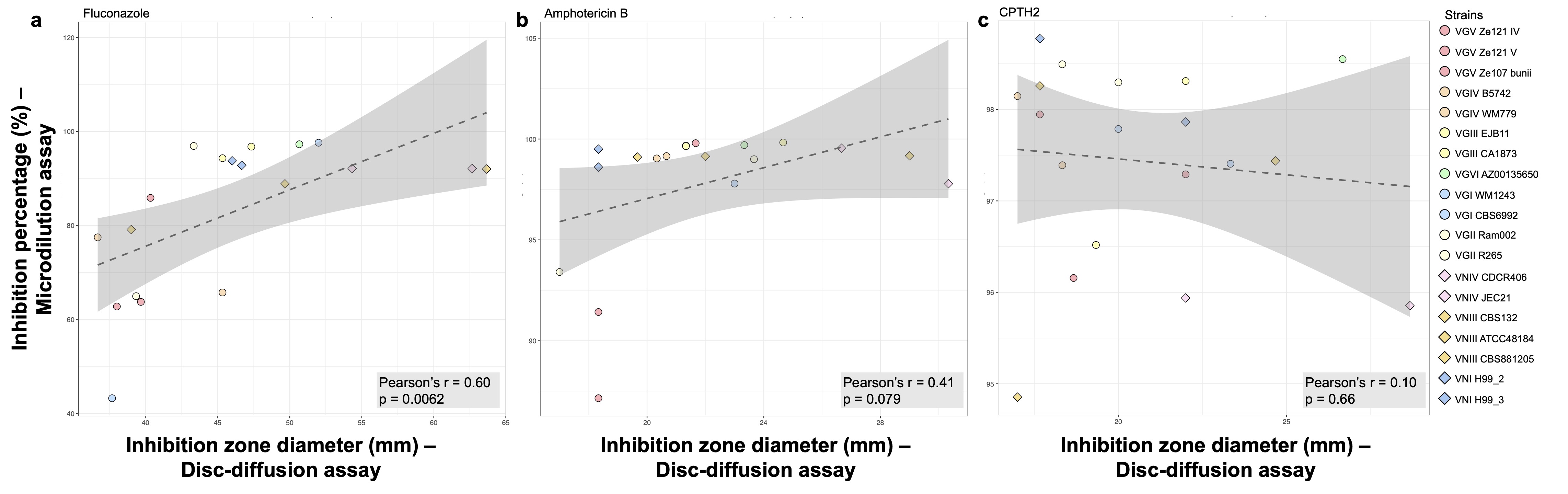

Supplement: Supplementary file 2 — Supplementary Material 2 [file 41598_2026_52566_MOESM2_ESM.jpg]

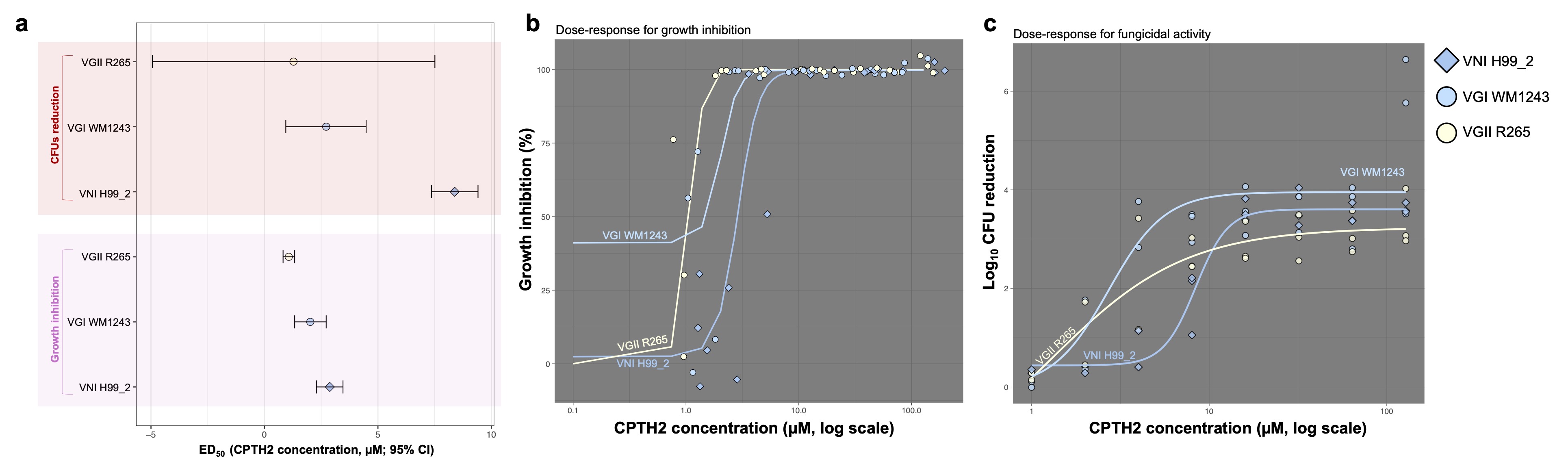

Supplement: Supplementary file 3 — Supplementary Material 3 [file 41598_2026_52566_MOESM3_ESM.jpg]

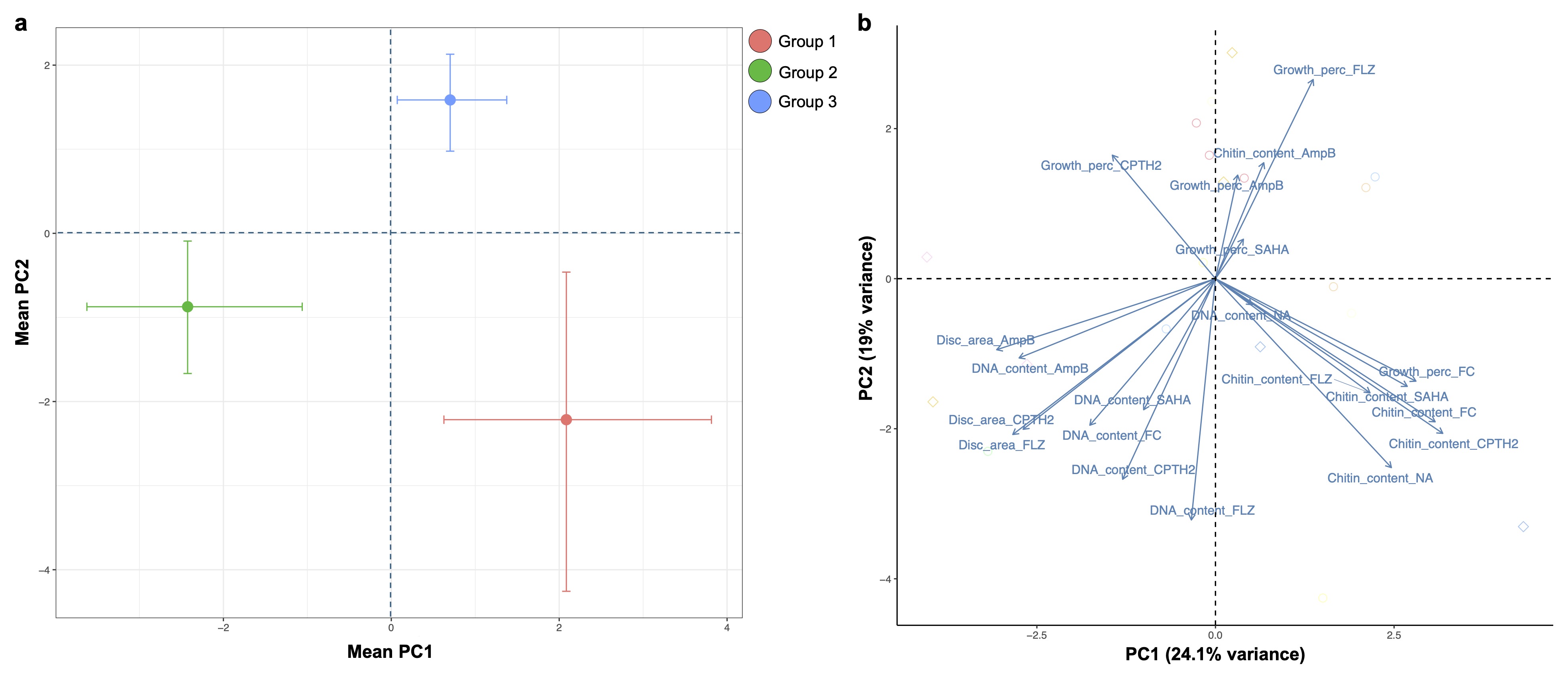

Supplement: Supplementary file 4 — Supplementary Material 4 [file 41598_2026_52566_MOESM4_ESM.jpg]

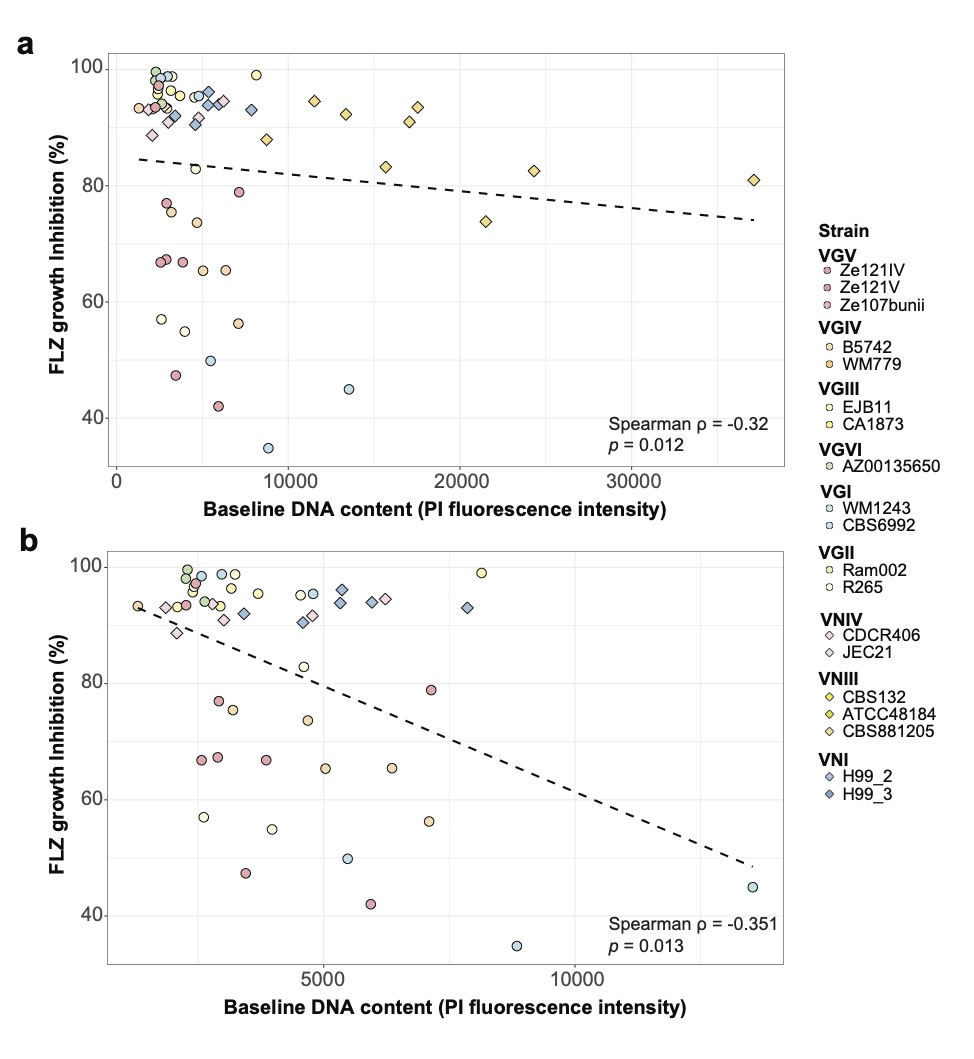

Supplement: Supplementary file 5 — Supplementary Material 5 [file 41598_2026_52566_MOESM5_ESM.jpg]
