# Supplementary material for: Antifungal-induced DNA dynamics and chitin remodelling across Cryptococcus spp. and the novel broad-spectrum anti-cryptococcal candidate CPTH2
Source: Sci Rep. 2026 May 19;16:22622. doi: 10.1038/s41598-026-52566-9 (PMC13381734; doi:10.1038/s41598-026-52566-9)
Supplement: Supplementary file 6 — Supplementary Material 6 [file 41598_2026_52566_MOESM6_ESM.docx]

**Supplementary information**

**Supplementary figures**

**Figure S1. Phenotypic classification of strains by microdilution assay based on *z-*score.** Scatter plots showing the strain inhibition (x-axis) versus robust *z-*score (y-axis) for (**a**) 5-FC, (**b**) FLZ, (**c**) AmpB, and (**d**) CPTH2. Data represent mean ± SD from three independent biological replicates. Each point corresponds to one strain. Mean percent inhibition was calculated as *100 – relative growth*. Robust *z*-scores were standardised to the drug specific cohort median and MAD across 19 strains. Point border colors indicate the classification: increased susceptibility (green), decreased susceptibility (orange), or indeterminate (grey). Dashed red lines indicate *z* thresholds (±0.8). Dashed blue lines denote ±5% margins around the cohort median. Strain classifications were assigned per drug as follows: increased if *z* ≥ 0.8 or mean inhibition ≥ 75^th^ percentile and ≥ median +5% growth inhibition; decreased if *z* ≤ -0.8 or mean ≤ 25^th^ percentile and ≤ median -5% growth inhibition, otherwise, indeterminate.

**Figure S2. Correlation between microdilution and disc-diffusion assays for antifungal susceptibility testing in *Cryptococcus.*** Scatter plots showing Pearson’s correlation between broth microdilution (inhibition percentage, y-axis) and disc-diffusion (inhibition zone diameter, x-axis) assays for (**a**) FLZ, (**b**) AmpB, and (**c**) CPTH2. Values were calculated from three independent biological replicates (data are expressed as mean ± SD). Each point represents one individual strain. Correlation coefficients (r) and associated *p* values are indicated in each panel. A strong correlation was observed for FLZ, a moderate correlation for AmpB and no significant correlation for CPTH2. These results indicates that FLZ susceptibility is consistently captured by both assays, supporting their reliability for antifungal susceptibility testing in *Cryptococcus.*

**Figure S3. Pharmacodynamic modelling of CPTH2 across three *Cryptococcus* lineages. a.** Estimated ED_50_ values (with 95% CI) for CPTH2, representing the concentrations required to cause growth inhibition (lower panel) and cell death (upper panel). **b.** Dose-response curves for CPTH2 using a log-logistic model (LL.4 for VNI H99_2 and VGI WM1243, LL.3 model for VGII R265). The curves illustrate the progressive growth inhibition with increasing concentrations of CPTH2 for the three lineages tested. **c.** Pharmacodynamic modelling of CPTH2 fungicidal activity using the LL.4 log-logistic model, highlighting its fungicidal effect across the tested lineages. Model fits are supported by significant ED_50_ values across all lineages (p < 0.0001). Values from three independent biological replicates are shown for each strain, with each point representing a single replicate measurement.

**Figure S4. Spearman’s correlation between baseline DNA content and FLZ susceptibility in *Cryptococcus* spp. a.** Scatterplot showing the relationship between baseline DNA content (measured under control conditions by flow cytometry) and FLZ growth inhibition (%) in *Cryptococcus* strains. Each point represents an individual strain and an individual replicate. A negative Spearman correlation was observed (Spearman ρ = -0.32, p = 0.012), indicating that strains with higher DNA content tend to exhibit reduced susceptibility to FLZ. The dashed line represents the linear regression trend. **b.** Scatterplot showing the same relationship but removing the VNIII strains. This produced a nearly identical result (Spearman ρ = -0.351, p = 0.013).

**Figure S5. PCA of phenotypic group separation and variable contributions. a.** Confidence intervals (CI) for phenotypic group centroids in PCA space. Each point represents the mean position of a phenotypic group centroid across 100 bootstrap iterations. Horizontal and vertical error bars indicate the 95% CI for PC1 and PC2, respectively. Although silhouette analysis yielded a modest average width of 0.288 (suggesting a weak to moderate separation), bootstrap intervals provide additional support for phenotypic grouping structure and variance stability. Colors indicate the phenotypic groups. **b.** PCA biplot showing the contribution of individual phenotypic variables to the variance captured in PC1 and PC2. Arrows represent the variables contributing, with their direction and length indicating the strength and orientation of each variable’s influence on the PCA space.

**Supplementary tables**

**Table S1. Modelling parameters for CPTH2-induced log_10_ CFU reduction:** LL.4 modelling was applied across the three *Cryptococcus* strains. Significant fungicidal activity was observed in VNI H99_2 and VGI WM1243. ED_50_, ED_90_ and ED_99_ values are shown with 95% CI.

|  | **VNI H99_2** | **VGI WM1243** | **VGII R265** |
| --- | --- | --- | --- |
| **Slope** | -4.77 (*p* = 0.06) | -2.75 (*p* = 0.67) | -1.14 (*p* = 0.3) |
| **Lower limit** | 0.43 (*p* = 0.01) | -0.02 (*p* = 0.98) | -2.1 (*p* = 0.76) |
| **Maximum effect** | 3.60 (*p* < 0.0001) | 3.95 (*p* < 0.0001) | 3.23 (*p* < 0.0001) |
| **ED_50_** | 8.38 µM (*p* < 0.0001, 95% CI, 7.36 - 9.41 µM) | 2.72 µM (*p* = 0.0048, 95% CI, 0.94 - 4.48 µM) | 1.28 µM (*p* = 0.66, 95% CI, -4.94 - 7.51 µM) |
| **ED_90_** | 13.29 µM (95% CI, 6.7 - 19.9 µM) | 6.03 µM (95% CI, -17.23 - 29.3 µM) | 8.74 µM (95% CI, -6.72 - 24.21 µM) |
| **ED_99_** | 21.95 µM (95% CI, -0.36 - 44.3) | 14.41 µM (95% CI, -102 - 130.8 µM) | 70.95 µM (95% CI, -192.28 - 334.19 µM) |

**Table** **S2. PCA contributors to variation in the phenotypic data**

| **Rank** | **Variable (PC1, 24.1%)** | **Loading (PC1)** | **Variable (PC2, 19%)** | **Loading (PC2)** |
| --- | --- | --- | --- | --- |
| 1 | Chitin_content_CPTH2 | 0.338 | DNA_content_FLZ | 0.385 |
| 2 | Chitin_content_5-FC | 0.326 | DNA_content_CPTH2 | 0.32 |
| 3 | Disc_area_AmpB | 0.325 | Growth_perc_FLZ | 0.318 |
| 4 | Disc_area_FLZ | 0.301 | Chitin_content_DMSO | 0.301 |
| 5 | Growth_perc_5-FC | 0.298 | Disc_area_FLZ | 0.248 |
| 6 | DNA_content_AmpB | 0.292 | Chitin_content_CPTH2 | 0.247 |
| 7 | Disc_area_CPTH2 | 0.286 | Disc_area_CPTH2 | 0.24 |
| 8 | Chitin_content_SAHA | 0.285 | DNA_content_5-FC | 0.234 |
| 9 | Chitin_content_DMSO | 0.261 | Chitin_content_5-FC | 0.228 |
| 10 | Chitin_content_FLZ | 0.229 | DNA_content_SAHA | 0.209 |
| 11 | DNA_content_5-FC | 0.186 | Growth_perc_CPTH2 | 0.197 |
| 12 | Growth_perc_CPTH2 | 0.153 | Chitin_content_AmpB | 0.185 |
| 13 | Growth_perc_FLZ | 0.145 | Chitin_content_FLZ | 0.182 |
| 14 | DNA_content_CPTH2 | 0.138 | Chitin_content_SAHA | 0.172 |
| 15 | DNA_content_SAHA | 0.106 | Growth_perc_AmpB | 0.165 |
| 16 | Chitin_content_AmpB | 0.071 | Growth_perc_5-FC | 0.163 |
| 17 | DNA_content_DMSO | 0.054 | DNA_content_AmpB | 0.126 |
| 18 | Growth_perc_SAHA | 0.041 | Disc_area_AmpB | 0.113 |
| 19 | DNA_content_FLZ | 0.035 | Growth_perc_SAHA | 0.063 |
| 20 | Growth_perc_AmpB | 0.033 | DNA_content_DMSO | 0.041 |

**Table S3. Clustering of strains after principal component analysis**

| **Lineage** | **Strain** | **Cluster ID** | **PC1 score** | **PC2 score** | **PC3 score** | **Phenotype** | **Origin** |
| --- | --- | --- | --- | --- | --- | --- | --- |
| VGV | Ze121IV | Group3 | -0.086 | 1.645 | -1.341 | Decreased susceptibility to AmpB | Environmental |
| VGV | Ze121V | Group3 | -0.267 | 2.075 | -1.528 | Decreased susceptibility to FLZ and AmpB | Environmental |
| VGV | Ze107bunii | Group3 | 0.403 | 1.340 | -0.534 | Decreased susceptibility to FLZ | Environmental |
| VGIV | B5742 | Group3 | 2.105 | 1.214 | 0.334 | Decreased susceptibility to FLZ and 5-FC | Clinical |
| VGIV | WM779 | Group3 | 1.652 | -0.107 | 0.819 | Decreased susceptibility to FLZ, 5-FC and CPTH2 | Veterinary |
| VGIII | EJB11 | Group2 | -0.172 | 0.214 | 1.414 | Decreased susceptibility to 5-FC, borderline increased susceptibility to FLZ |  |
| VGIII | CA1873 | Group1 | 1.503 | -4.256 | -0.512 | Decreased susceptibility to 5-FC, increased susceptibility to AmpB |  |
| VGVI | AZ00135650 | Group2 | -3.186 | -2.302 | 0.192 | Increased susceptibility to FLZ, 5-FC, and CPTH2 | Clinical |
| VGI | WM1243 | Group2 | -0.690 | -0.670 | 0.850 | Increased susceptibility to FLZ and CPTH2 | Clinical |
| VGI | CBS6992 | Group3 | 2.234 | 1.357 | -1.713 | Decreased susceptibility to FLZ | Clinical |
| VGII | Ram002 | Group3 | -0.050 | 2.360 | 0.708 | Decreased susceptibility to FLZ and 5-FC | Clinical |
| VGII | R265 | Group1 | 1.904 | -0.461 | -0.532 | Decreased susceptibility to 5-FC and AmpB | Clinical |
| VNIV | CDCR406 | Group2 | -2.638 | -1.143 | 2.218 | Increased susceptibility to FLZ and AmpB | Clinical |
| VNIV | JEC21 | Group2 | -4.039 | 0.287 | -1.249 | Increased susceptibility to FLZ, AmpB and CPTH2 | Clinical |
| VNIII | CBS132 | Group2 | -3.954 | -1.641 | -2.595 | Increased susceptibility to 5-FC, FLZ, AmpB and CPTH2 |  |
| VNIII | ATCC48184 | Group3 | 0.233 | 3.012 | 0.049 | Decreased susceptibility to FLZ, 5-FC and CPTH2 | Environmental |
| VNIII | CBS881205 | Group3 | 0.113 | 1.289 | 0.979 | Indeterminate | Clinical |
| VNI | H99_2 | Group1 | 0.627 | -0.908 | 4.173 | Decreased susceptibility to 5-FC and AmpB | Environmental |
| VNI | H99_3 | Group1 | 4.310 | -3.305 | -1.733 | Decreased susceptibility to 5-FC and AmpB | Environmental |

**Table S4.** Bootstrapped means and 95% CIs for the first three PC scores (PC1-PC3) across phenotypic groups. Values are based on 1000 bootstrap resampling. The values summarise the central tendency and variability of strain positions in PCA space, supporting group separation observed in the analysis.

| **Cluster** | **PC** | **Mean** | **Lower 95% CI** | **Upper 95% CI** |
| --- | --- | --- | --- | --- |
| Group1 | PC1 | 2.09 | 0.63 | 3.81 |
| Group1 | PC2 | -2.22 | -4.26 | -0.46 |
| Group1 | PC3 | 0.30 | -1.48 | 4.17 |
| Group2 | PC1 | -2.43 | -3.63 | -1.06 |
| Group2 | PC2 | -0.87 | -1.67 | -0.09 |
| Group2 | PC3 | 0.15 | -1.25 | 1.46 |
| Group3 | PC1 | 0.70 | 0.07 | 1.38 |
| Group3 | PC2 | 1.59 | 0.98 | 2.13 |
| Group3 | PC3 | -0.24 | -0.96 | 0.44 |

**Table S5. *Cryptococcus* strains used in this study**

| **Phylogenetic lineage** | **Strain** | **Original name** | **Source** | **Lab origin** |
| --- | --- | --- | --- | --- |
| **VNI** | Cn_H99_3 | H99 Wildtype Zaragoza | Pigeon, New York | Professor Robin May |
|  | Cn_H99_2 | H99 Newcastle | Pigeon, New York |  |
| **VNIII** | Cn_CBS881205 | 881205-II | HIV |  |
|  | Cn_ATCC48184 | ATCC 48184 | Bird droppings, Japan, Takeo |  |
|  | Cn_CBS132 | CBS132 |  |  |
| **VNIV** | Cn_JEC21 | JEC21 | AIDS, New York |  |
|  | Cn_CDCR406 | CDCR406 | Vancouver outbreak |  |
| **VGI** | Cg_CBS6992 | CBS6992 | Man, USA |  |
|  | Cg_WM1243 | WM1243 | Clinical, HIV |  |
| **VGII** | Cg_R265 | R265(b) | Clinical, Vancouver |  |
|  | Cg_Ram002 | Ram002 | Australia |  |
| **VGIII** | Cg_CA1873 | CA1873 |  |  |
|  | Cg_EJB11 | EJB11 |  |  |
| **VGIV** | Cg_WM779 | WM779 | Veterinary, South Africa |  |
|  | Cg_B5742 | B5742 | CSF, HIV, India |  |
| **VGV** | Cg_Ze107bun ii | Ze107bun ii |  | Professor Mat Fisher |
|  | Cg_Ze121 V | Ze121 V |  |  |
|  | Cg_Ze121 IV | Ze121 IV |  |  |
| **VGVI** | Cg_AZ00135650 | AZ00135650 | Clinical, USA/AZ, Isolated in 2021 from CSF | Professor David Engelthaler |
